# Augmented AMPK activity inhibits cell migration by phosphorylating the novel substrate Pdlim5

**DOI:** 10.1038/ncomms7137

**Published:** 2015-01-30

**Authors:** Yi Yan, Osamu Tsukamoto, Atsushi Nakano, Hisakazu Kato, Hidetaka Kioka, Noriaki Ito, Shuichiro Higo, Satoru Yamazaki, Yasunori Shintani, Ken Matsuoka, Yulin Liao, Hiroshi Asanuma, Masanori Asakura, Kazuaki Takafuji, Tetsuo Minamino, Yoshihiro Asano, Masafumi Kitakaze, Seiji Takashima

**Affiliations:** 1 Department of Medical Biochemistry, Osaka University Graduate School of Medicine, 2-2 Yamadaoka, Suita, Osaka 565-0871, Japan; 2 Depertment of Clinical Research and Development, National Cerebral and Cardiovascular Center Research Institute, Suita, Osaka 565-8565, Japan; 3 Department of Cardiovascular Medicine, Osaka University Graduate School of Medicine, 2-2 Yamadaoka, Suita, Osaka 565-0871, Japan; 4 Department of Cell Biology, National Cerebral and Cardiovascular Center Research Institute, Suita, Osaka 565-8565, Japan; 5 Department of Cardiology, Nanfang Hospital, Southern Medical University, 1838 North Guangzhou Avenue, 510515 Guangzhou, China; 6 Center for Research Education, Osaka University Graduate School of Medicine, 2-2 Yamadaoka, Suita, Osaka 565-0871, Japan; 7 Japan Science and Technology Agency-Core Research for Evolutional Science and Technology (CREST), Kawaguchi 332-0012, Japan

## Abstract

Augmented AMP-activated protein kinase (AMPK) activity inhibits cell migration, possibly contributing to the clinical benefits of chemical AMPK activators in preventing atherosclerosis, vascular remodelling and cancer metastasis. However, the underlying mechanisms remain largely unknown. Here we identify PDZ and LIM domain 5 (Pdlim5) as a novel AMPK substrate and show that it plays a critical role in the inhibition of cell migration. AMPK directly phosphorylates Pdlim5 at Ser177. Exogenous expression of phosphomimetic S177D-Pdlim5 inhibits cell migration and attenuates lamellipodia formation. Consistent with this observation, S177D-Pdlim5 suppresses Rac1 activity at the cell periphery and displaces the Arp2/3 complex from the leading edge. Notably, S177D-Pdlim5, but not WT-Pdlim5, attenuates the association with Rac1-specific guanine nucleotide exchange factors at the cell periphery. Taken together, our findings indicate that phosphorylation of Pdlim5 on Ser177 by AMPK mediates inhibition of cell migration by suppressing the Rac1-Arp2/3 signalling pathway.

## Abstract (web summary)

Augmented AMP-activated protein kinase (AMPK) activity inhibits cell migration through an unknown mechanism. Here, Yan *et al.* show that AMPK phosphorylates the novel substrate PDZ and LIM domain 5 (Pdlim5), and that phosphomimetic Pdlim5 impairs cell migration by disrupting the Rac1-Arp2/3 signalling pathway.

## Graphical abstract

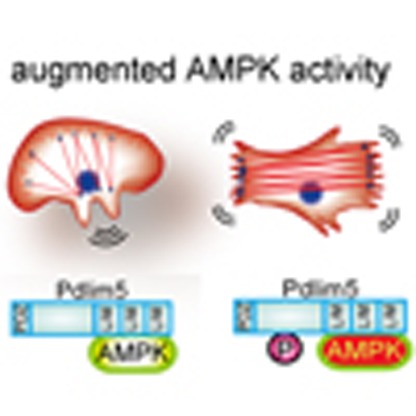

AMP-activated protein kinase (AMPK), generally considered an energy sensor kinase, requires AMP for activation^1^. Recently, a growing body of evidence has revealed that AMPK also plays a key role in the establishment of cell polarity and motility^2^,^3^. We previously reported that AMPK regulates cell migration by controlling microtubule dynamics through phosphorylation of a cytoplasmic linker protein-170 (CLIP-170)^4^. Moreover, recent studies have implicated AMPK in the regulation of actin cytoskeleton dynamics and reorganization at the plasma membrane^5^,^6^. Thus, AMPK is predicted to regulate cell migration by controlling both microtubule and actin-filament dynamics.

Cell migration is a physically integrated molecular process that begins with dynamic polarization and formation of lamellipodia, membrane protrusions at the leading edges of cells^7^. Rac1, a Rho-family small GTPase, is a key upstream regulator of actin dynamics and organization, and is necessary for the formation of persistent lamellipodia leading to directional cell migration^8^,^9^. Once Rac1 is activated by guanine nucleotide exchange factors (GEFs) at the leading edge, the activated form (GTP-bound Rac1) recruits a complex containing its downstream effector Wiskott–Aldrich Syndrome protein family verprolin homologous to the plasma membrane, leading in turn to activation of the actin-related protein 2/3 (Arp2/3) complex^10^,^11^. Activated Arp2/3 complex functions as an efficient nucleator^10^,^11^ to organize the branched actin-filament network involved in formation of lamellipodia, a critical step in cell migration.

Some drugs in clinical use have the potential to indirectly activate AMPK. These compounds have been convincingly shown to prevent atherosclerosis, vascular remodelling, and tumour invasion and metastasis^12^,^13^,^14^,^15^,^16^,^17^, processes in which dysregulated cell migration contributes to the development and progression of diseases. Accordingly, the clinically beneficial effects of chemical AMPK activators can be partially attributed to inhibition of cell migration via augmentation of AMPK activity. However, the mechanisms by which augmented AMPK activity inhibits cell migration remain largely unknown.

In this study, we identified PDZ and LIM domain 5 (Pdlim5)^18^ as a novel substrate of AMPK; Pdlim5 is directly phosphorylated by AMPK at Ser177. This phosphorylation results in displacement of Rho GEF 6 (Arhgef6), a Rac1/Cdc42-specific GEF and also known as p21-activated kinase-interacting exchange factor-αPIX, from the leading edge of the cell by disrupting the association between Pdlim5 and Arhgef6. Displacement of Arhgef6 suppresses Rac1 activity and the disappearance of Arp2/3 complex from the cell periphery, leading to defects in lamellipodia formation and inhibition of directional cell migration. We propose that Pdlim5 is the main signalling molecule that regulates cell migration in the context of augmented AMPK activity.

## Results

### Pdlim5 is phosphorylated at Ser177 by AMPK

**Figure 1:**
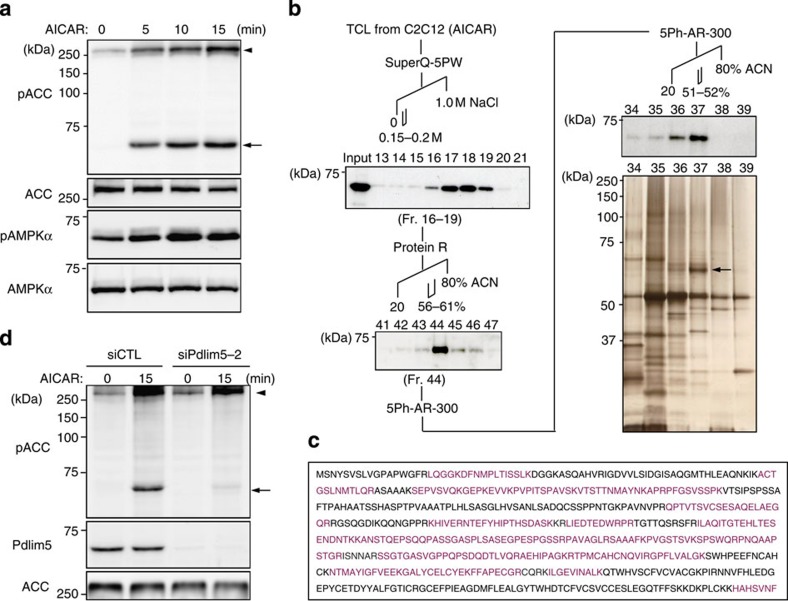
Pdlim5 is a substrate of AMPK. (**a**) C2C12 cells were stimulated with AICAR (2 mM) 12 h after serum starvation. Total cell lysates (TCLs) were harvested 0, 5, 10 and 15 min after stimulation and subjected to immunoblotting with anti-pACC antibody. In addition to the predicted band for pACC (arrowhead), a cross-reacting band, p64 (arrow), was detected in a time-dependent manner after AICAR treatment. (**b**) A schematic representation of the purification and identification of p64. TCLs from C2C12 cells treated with AICAR were subjected to three-step column chromatography (superQ-5PW, protein R and 5Ph-AR-300). p64 was detected using the anti-pACC antibody in each step. Images were obtained by immunoblotting and silver staining. The identified band (arrow in silver-stained gel), fractionated by reverse-phase HPLC (5Ph-AR-300), was excised and analysed by mass spectrometry. (**c**) Amino acid sequence of Pdlim5. Sequence analysis by MALDI-Qq-TOF MS/MS revealed the target protein to be Pdlim5. Matching amino acids are shown in magenta letters. (**d**) C2C12 cells treated with siRNA (siCTL or siPdlim5-2) were stimulated with AICAR and TCLs were subjected to immunoblotting with pACC and Pdlim5 antibodies (Ab229-2). Arrowhead and arrow denote pACC and p64, respectively.

Our group has worked on AMPK for many years. To estimate the AMPK activity level *in vivo*, we often monitor phospho-acetyl-CoA carboxylase (pACC), a well-known substrate of AMPK whose level generally reflects the AMPK activity^4^. When we treated C2C12 cells with AMPK activators such as 5-aminoimidazole-4-carboxyamide ribonucleoside (AICAR), A-769662 and 2-deoxy-D-glucose (2-DG), we serendipitously observed reproducible and specific induction of a protein with an apparent molecular mass of 64 kDa (p64) that cross-reacted with a commercially available antibody against pACC (Fig. 1a and Supplementary Fig. 1a,b, respectively). Three-step column chromatography followed by mass-spectrometric analysis revealed that p64 was likely to be Pdlim5 (ref. 19; Fig. 1b,c). To confirm this, we generated three polyclonal antibodies against mouse Pdlim5 (Supplementary Fig. 1c) and designed two different small interfering RNAs (siRNAs) against the *Pdlim5* messenger RNA (Supplementary Fig. 1d). When we treated C2C12 cells with siPdlim5, the p64 band disappeared (Fig. 1d). Therefore, we concluded that p64 is indeed Pdlim5. Furthermore, as the p64 band, probably representing a phosphorylated form of Pdlim5, was detected exclusively after AMPK activation, we speculated that Pdlim5 is a substrate of AMPK. Pdlim5, also known as Enigma homolog protein, is an α-actinin-binding protein that possesses a PDZ domain at its amino terminus and three LIM motifs at its carboxy terminus^19^. Pdlim5 anchors to the actin cytoskeleton via its PDZ domain and recruits LIM-associated proteins to actin filaments^20^. To narrow down the location of the phosphorylation site, we transfected wild-type (WT) Pdlim5 or deletion mutants (Supplementary Fig. 2a) into HEK293T cells, and then treated the transfectants with AICAR. A mutant truncated after N184 cross-reacted with the pACC antibody following AMPK activation (Supplementary Fig. 2b), whereas a mutant truncated after N160 did not, indicating that the phosphorylation site resides within the N-terminal segment of Pdlim5 between Ala161 and Asn184.

**Figure 2:**
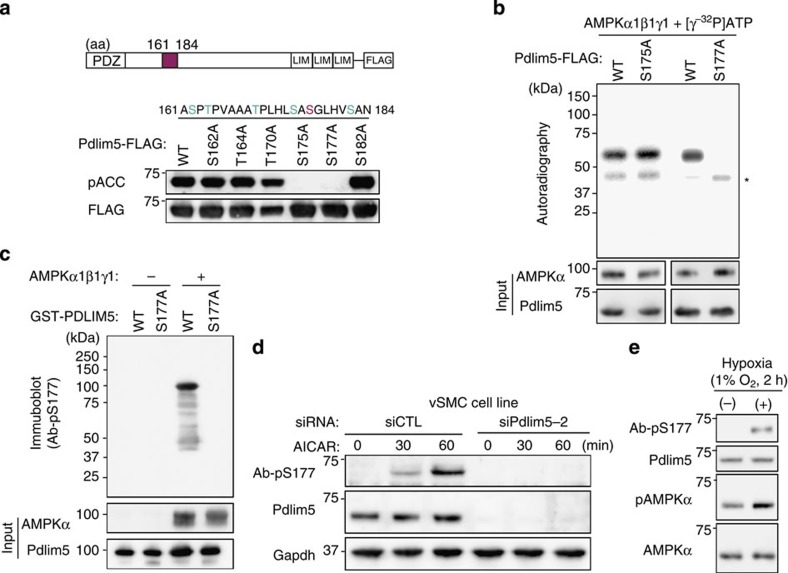
Pdlim5 is directly phosphorylated at Ser177 by AMPK. (**a**) Diagrams of point-mutated Pdlim5. HEK293T cells were transiently expressed with each point-mutant of cFLAG-tagged Pdlim5 and treated with AICAR (2 mM) for 15 min. Proteins purified on anti-FLAG M2 agarose were subjected to immunoblotting with anti-pACC or anti-FLAG antibody. (**b**) *In vitro* assay for AMPK phosphorylation. Recombinant cFLAG-tagged Pdlim5 (WT, S175A or S177A) from HEK293T cells was incubated with baculovirus-expressed recombinant AMPK in the presence of [γ-^32^P]ATP and then subjected to autoradiography. Asterisk denotes bands of α-subunit of recombinant AMPK. (**c**) *In vitro* phosphorylation assay by AMPK. Recombinant GST-tagged Pdlim5 proteins (WT and S177A) were incubated with or without recombinant AMPK and then subjected to immunoblotting with Ab-pS177. (**d**) vSMCs transfected either with siCTL or siPdlim5-2 were treated with AICAR (2 mM) for the indicated times, and TCLs were subjected to immunoblotting with Ab-pS177. (**e**) vSMCs were exposed to physiological hypoxia (1% O_2_ for 2 h). TCLs were subjected to immunoblotting with the indicated antibodies.

Next, we introduced Ser-to-Ala or Thr-to-Ala point mutations into this putative phosphorylation segment (Fig. 2a). Both the S175A and S177A mutants lost cross-reactivity with pACC antibody (Fig. 2a). Next, we performed *in vitro* phosphorylation assays on recombinant FLAG-tagged Pdlim5 (WT, S175A and S177A). The results revealed that incorporation of [γ-^32^P]ATP into Pdlim5 was inhibited only in the S177A mutant, indicating that Ser177 is the unique phosphorylation site of Pdlim5 (Fig. 2b). We confirmed direct phosphorylation of Pdlim5 at Ser177 by AMPK by *in vitro* phosphorylation assay using recombinant glutathione *S*-transferase (GST)-fused Pdlim5 (WT or S177A) expressed in *Escherichia coli* (Supplementary Fig. 2c,d), and subsequently by using an antibody we generated (Supplementary Fig. 2e) against Ser177-phosphorylated Pdlim5 (Ab-pS177) (Fig. 2c). The amino-acid sequence surrounding Ser177 matches the consensus sequence for AMPK phosphorylation sites (Supplementary Fig. 2f) and also exhibits a high similarity with the phosphorylation site of ACC (Supplementary Fig. 2f). Furthermore, Ser177, but not Ser175, is highly conserved only in mammals (Supplementary Fig. 3). Next, we confirmed that endogenous Pdlim5 is phosphorylated at Ser177 on AMPK activation *in vivo* in vascular smooth muscle cells (vSMCs) (Fig. 2d and Supplementary Fig. 4). Finally, we investigated whether Pdlim5 is phosphorylated in response to an acute physical stimulus. Hypoxia (1% O_2_ for 2 h) activated AMPK and induced Ser177-phosphorylation of Pdlim5 (Fig. 2e). Together, these data indicated that augmented AMPK activity induces Ser177 phosphorylation of Pdlim5 both *in vitro* and *in vivo*.

### Ser177 phosphorylation of Pdlim5 inhibits cell migration

**Figure 3:**
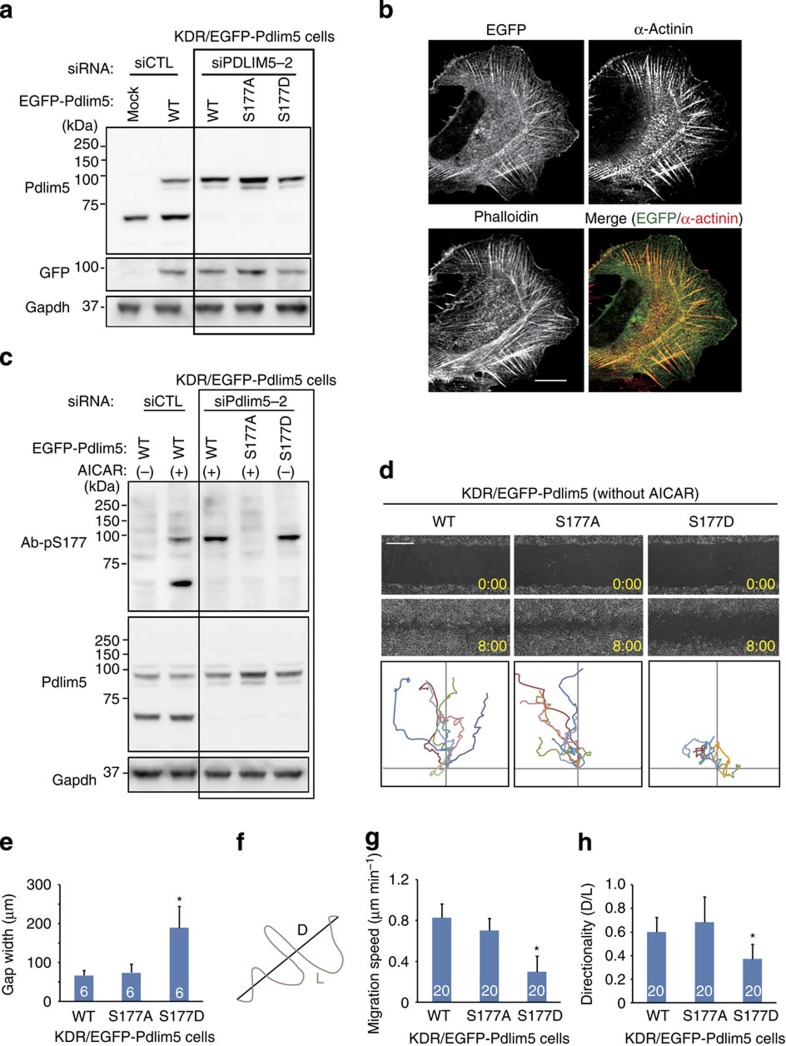
Ser177 phosphorylation of Pdlim5 by AMPK inhibited directional migration of vSMCs. (**a**) Establishment of the KDR system for Pdlim5 in vSMCs. vSMCs were transfected with either siCTL or siPdlim5-2. siPdlim5-2-resistant EGFP-Pdlim5 (WT, S177A or S177D) was added back by adenoviral-mediated gene delivery. TCLs were subjected to immunoblotting. (**b**) GFP and immunostained images of KDR/EGFP-WT-Pdlim5 cells stained with a α-actinin antibody and phalloidin. Scale bar, 10 μm. (**c**) KDR/EGFP-Pdlim5 (WT and S177A) cells were treated with AICAR for 60 min, whereas KDR/EGFP-S177D-Pdlim5 cells were not treated with AICAR. TCLs from each cell were subjected to immunoblotting. (**d**) Scratch assay of KDR/EGFP-Pdlim5 cells. Phase-contrast microscopy images of KDR/Pdlim5 cells (WT, S177A and S177D) before and 8 h after scratching in the absence of AICAR. The bottom row of each panel shows analysis of migration paths over 8 h. The origins of migration of each cell were superimposed at [0, 0]. Scale bar, 0.5 mm. (**e**) Bar graph showing the gap width 8 h after scratching (from **d**). (**f**) Demonstration of path length (L) and displacement (D) for calculation of migration velocity and directionality. (**g**) Bar graph showing migration speed of each cell (from **d**). (**h**) Bar graph showing migration directionality of each cell (from **d**). Numbers in the bars indicate *n*. Data are representative of means±s.e.m. from three independent experiments. Significance of differences between series of results was assessed using one-way analysis of variance, followed by a *post-hoc* comparison with Dunnett’s method for multiple comparisons. *P<0.01 compared with WT.

To accurately assess the functions of Ser177 phosphorylation of Pdlim5 by AMPK, it is essential to use a system that eliminates any effects associated with AMPK activation other than Ser177 phosphorylation. For this purpose, we established the knockdown-rescue (KDR) system in vSMCs by depleting endogenous Pdlim5 and simultaneously expressing siPDLIM-resistant enhanced green fluorescent protein (EGFP)-tagged Pdlim5 (Fig. 3a). For this purpose, we treated cells with siRNA oligonucleotides targeting the 3′-untranslated region mRNA of mouse Pdlim5 (siPdlim5-2) and infected them with adenovirus encoding EGFP-WT-, EGFP-S177A- or EGFP-S177D-Pdlim5, yielding cells we designated KDR/WT-, KDR/S177A- or KDR/S177D-Pdlim5, respectively. This KDR system successfully replaced endogenous Pdlim5 with EGFP-Pdlim5 at physiological expression levels in vSMCs (Fig. 3a). Exogenously expressed EGFP-Pdlim5 co-localized with α-actinin and F-actin structures (Fig. 3b), a pattern similar to that of endogenous Pdlim5 (Supplementary Fig. 5), although EGFP-Pdlim5 appeared in the cytoplasm at much higher levels than the endogenous protein. Importantly, EGFP-WT-Pdlim5 was phosphorylated by AMPK in the same manner as endogenous Pdlim5, whereas EGFP-S177A-Pdlim5, an unphosphorylatable mutant, was not phosphorylated (Fig. 3c). EGFP-S177D-Pdlim5, a phosphomimetic mutant, was recognized by the Ab-pS177 antibody even in the absence of AICAR stimulation (Fig. 3c). This result indicates that phosphorylated WT-Pdlim5 and the S177D-Pdlim5 mutant are structurally similar. Thus, even in the absence of AMPK activators, exogenously expressed EGFP-S177D-Pdlim5 behaved similar to endogenous Pdlim5 phosphorylated by AMPK. It is particularly noteworthy that the KDR system can exclude any effects associated with AMPK activation other than Ser177 phosphorylation of Pdlim5.

AMPK activation inhibits cell migration; to investigate whether this effect is mediated by Pdlim5 phosphorylation at Ser177, we performed a scratch assay using the KDR/vSMC system in the absence of AMPK activators. KDR/S177D-Pdlim5 cells exhibited a marked delay in scratch closure relative to both KDR/WT- and KDR/S177A-Pdlim5 cells (Fig. 3d,e and Supplementary Movie 1). By tracking the movement of individual cells, we could calculate the path length (L) and displacement (D) of individual cells (Fig. 3f). KDR/S177D-Pdlim5 cells exhibited a lower migration speed (defined as L/total trajectory time) and less directionality (defined as D/L) than KDR/WT- and KDR/S177A-Pdlim5 cells (Fig. 3g,h). Next, we performed a scratch assay in the presence of AMPK activator (Supplementary Fig. 6). Compared with KDR/WT- and KDR/S177D-Pdlim5 cells, the degree of cell migratory inhibition was lower in KDR/S177A-Pdlim5 cells (Supplementary Fig. 6). Furthermore, we examined single-cell migration in the absence of AMPK activators. Consistent with the results of the scratch assay, KDR/S177D-Pdlim5 cells exhibited impaired cell migration (Supplementary Fig. 7a,b and Supplementary Movie 2).

**Figure 4:**
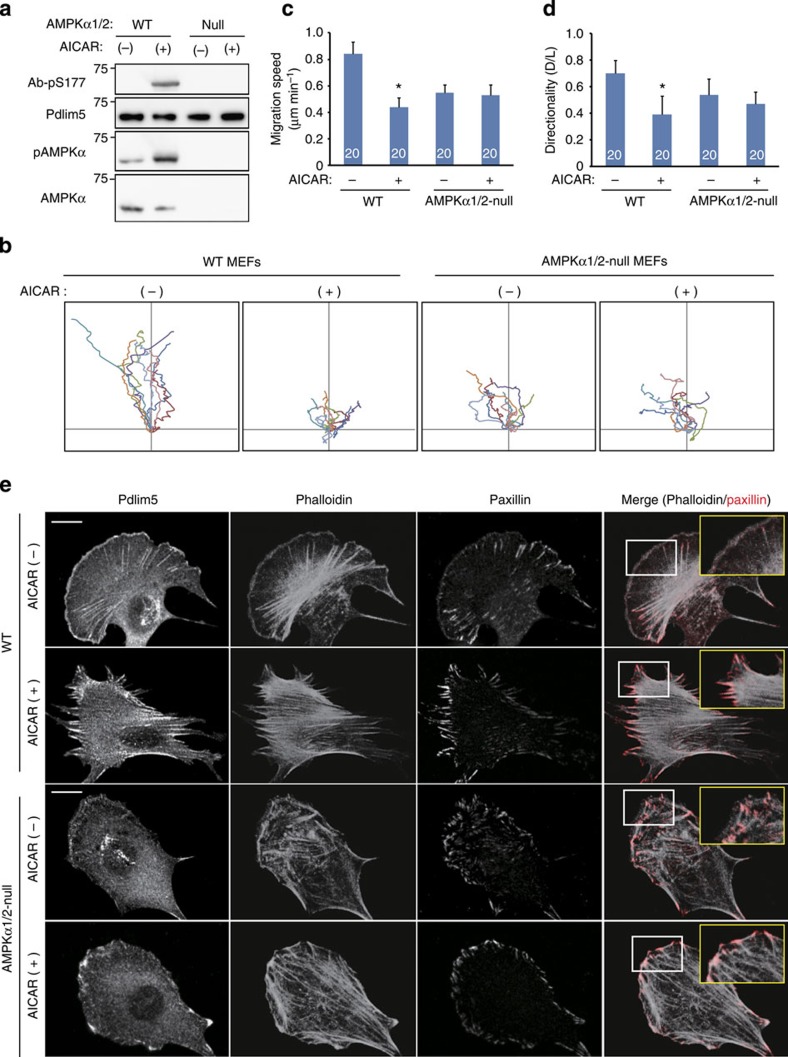
Pdlim5 phosphorylation was blocked in AMPKα1/2-null MEFs. (**a**) WT or AMPKα1/2-null MEFs were stimulated with AICAR (1 mM) for 15 min. TCLs were subjected to immunoblotting with indicated antibodies. (**b**) Phase-contrast images of WT-MEFs and AMPKα1/2-null MEFs were collected during the scratch assay in the presence or absence of AICAR (1 mM). Each panel shows analysis of migration paths over 8 h. The origins of migration of each cell were superimposed at [0, 0]. (**c**) Bar graph showing the migrating speed of each cell (from **b**). (**d**) Bar graph showing migration directionality of each cell (from **b**). Numbers in the bars indicate *n*. Data are representative of means±s.e.m. from three independent experiments. Significance of differences between series of results was assessed using one-way analysis of variance, followed by a *post-hoc* comparison with Dunnett’s method for multiple comparisons. *P<0.01 compared with WT without AICAR treatment. (**e**) Immunostained images of WT-MEFs and AMPKα1/2-null MEFs in the presence or absence of AICAR (1 mM). These cells were stained with a Pdlim5 antibody, phalloidin and anti-paxillin antibody. Magnified images outlined by yellow squares show the areas outlined by white squares. Scale bar, 10 μm.

Next, we examined the AMPK specificity of the phenotypes observed in KDR cells using AMPKα1^−/−^α2^−/−^ mouse embryonic fibroblasts (AMPKα-null MEFs). First, we confirmed that Pdlim5 phosphorylation by AMPK activators was completely blocked in AMPKα-null MEFs (Fig. 4a and Supplementary Fig. 8). Moreover, a scratch assay confirmed that AMPK activator-induced cell migratory inhibition was abolished in AMPKα-null MEFs (Fig. 4b–d and Supplementary Movie 3). For further confirmation, we constructed a Pdlim5 knockout vSMC (Pdlim5^−/−^ vSMC) line by genome editing, using the CRISPR/Cas9 system (Supplementary Fig. 9), and established a knockout and rescue cell system by expressing EGFP-Pdlim5 (WT, S177A or S177D) at physiological level by adenovirus transduction of knockout cells (Supplementary Fig. 10a). When the knockout and rescue cells were subjected to a cell migration assay (Supplementary Fig. 10b–d), the results were consistent with those obtained with the KDR cells. Together, these results indicated that Ser177 phosphorylation of Pdlim5 by AMPK inhibits cell migration.

### Ser177 phosphorylation altered actin architectures

**Figure 5:**
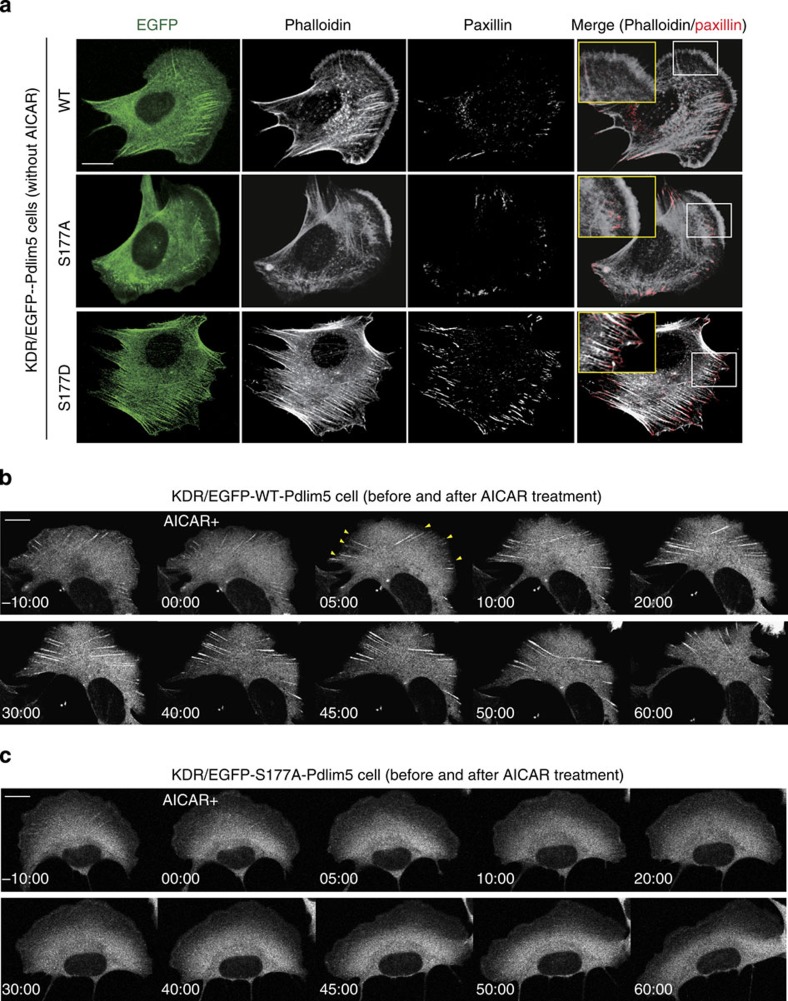
Ser177 phosphorylation of Pdlim5 causes reorganization of lamellipodia, stress fibres and focal adhesions. (**a**) GFP images (left) and immunostained images of KDR/EGFP-Pdlim5 cells (WT, S177A and S177D). Cells were stained with phalloidin and anti-paxillin antibody, to visualize actin microfilaments and focal adhesions, respectively. Magnified images outlined by yellow squares show the area outlined by the white squares. Scale bar, 10 μm. (**b**,**c**) Sequential GFP images of KDR/EGFP-WT-Pdlim5 (**b**) or KDR/EGFP-S177A-Pdlim5 (**c**) cells before and after AICAR stimulation (2 mM). Yellow arrowheads represent dorsal stress fibres growing from the opposite side after AICAR treatment of KDR/EGFP-WT-Pdlim5 cells. Scale bar, 10 μm.

To elucidate the mechanism by which Ser177 phosphorylation of Pdlim5 inhibits cell migration, we monitored morphological changes in KDR cells. In all three types of KDR cells, EGFP-Pdlim5 protein co-localized with F-actin at the cell periphery and on stress fibres (Fig. 5a), indicating that co-localization of Pdlim5 with actin was not influenced by the phosphorylation state of Ser177. However, KDR/WT- and KDR/S177A-Pdlim5 cells displayed smooth lamellipodia-like edges, whereas KDR/S177D-Pdlim5 cells exhibited attenuated lamellipodia formation and jagged edges with excessive filopodia-like protrusions and ventral stress fibres (Fig. 5a and Supplementary Fig. 11a). In addition, both KDR/WT- and KDR/S177A-Pdlim5 cells contained small and scattered spots of focal adhesions at the junction between the lamellipodia and lamella; by contrast, in KDR/S177D-Pdlim5 cells, focal adhesions were displaced to the edge of the cell and significantly enlarged in size (Fig. 5a and Supplementary Fig. 11b). To determine whether the morphological changes observed in KDR/S177D-Pdlim5 cells were related to Ser177 phosphorylation of Pdlim5, we performed time-lapse imaging before and after treatment with AMPK activator (Fig. 5b,c). In KDR/WT-Pdlim5 cells, but not in KDR/S177A-Pdlim5 cells, AMPK activation induced defective lamellipodia formation and promoted expansion of the EGFP signals from the side opposite the lamellae towards the cell centre (Fig. 5b,c and Supplementary Movie 4), a pattern resembling the growth of dorsal stress fibres. This morphological change was similar to that observed in KDR/S177D-Pdlim5 cells. Furthermore, AMPK activation induced defective lamellipodia and enhanced stress fibre formation in WT-MEFs, but not AMPKα-null MEFs (Fig. 4e). These findings suggested that Ser177 phosphorylation of Pdlim5 by augmented AMPK activity induced attenuation of lamellipodia and promoted formation of stress fibres from the cell periphery. Furthermore, these observations suggested that morphological changes of the actin architecture were initiated near the cell periphery.

### Ser177 phosphorylation altered Arp2/3 complex localization

**Figure 6:**
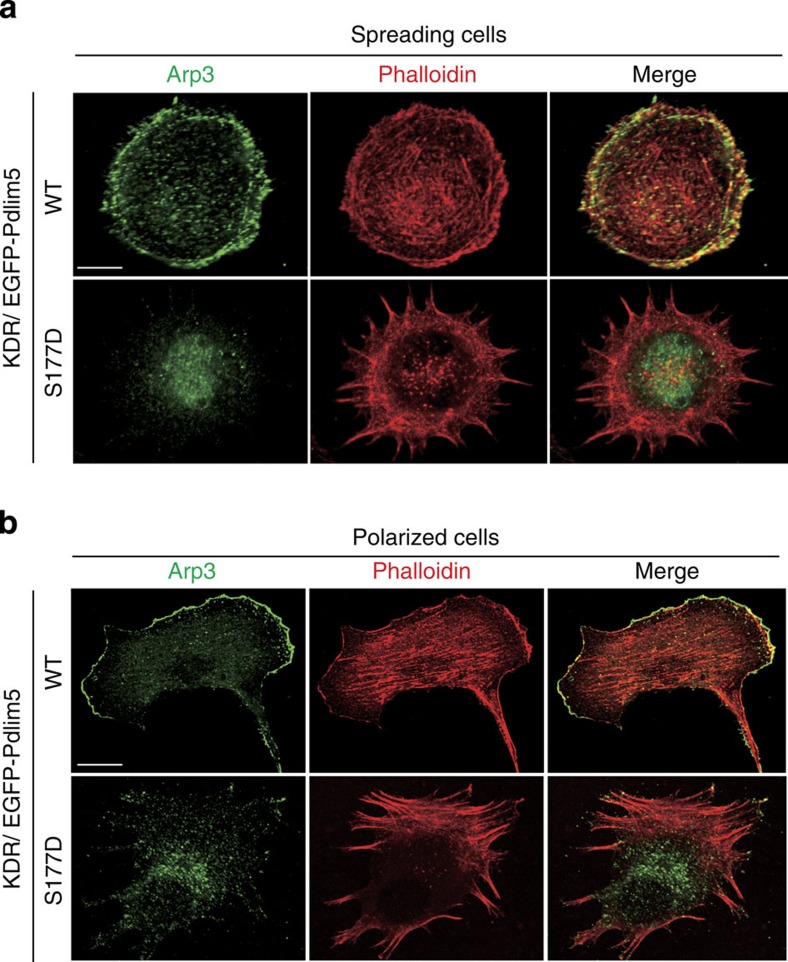
Ser177 phosphorylation of Pdlim5 leads to altered Arp2/3 complex localization. (**a**,**b**) Immunostaining of spreading (**a**) or polarized (**b**) KDR/EGFP-Pdlim5 cells. Cells were fixed and stained with an Arp3 antibody and phalloidin. Scale bars, 10 μm.

Accordingly, we focused on the Arp2/3 complex, because it is one of the major actin nucleators at the cell periphery and plays a key role in lamellipodia formation^10^. Expression levels of Arp2/3 complex were comparable among the various types of KDR cells (Supplementary Fig. 12a). However, the intracellular localization of Arp2/3 complex was markedly altered in both spreading and polarized cells (Fig. 6). In spreading cells, KDR/WT-Pdlim5 cells exhibited highly uniform lamellipodia throughout the cell edge, where Arp3 predominantly localized (Fig. 6a). By contrast, KDR/S177D-Pdlim5 cells exhibited filopodia-like protrusions instead of lamellipodia, and localization of Arp3 shifted from the cell edge to a cytoplasmic distribution (Fig. 6a). A similar pattern was observed in polarized cell: Arp3 localized in actin-rich lamellipodia in KDR/WT-Pdlim5 cells, but was distributed diffusely throughout the cytoplasm in KDR/S177D-Pdlim5 cells (Fig. 6b). These findings indicated that Ser177 phosphorylation of Pdlim5 impairs the function of the Arp2/3 complex by altering the localization of the complex from the cell edge to the cytoplasm. Consistent with this, vSMCs defective for Arp2/3 complex due to knockdown of the Arpc2 subunit exhibited a phenotype very similar to that of DR/S177D-Pdlim5 cells (Supplementary Fig. 12b,c). On the other hand, mammalian diaphanous (mDia), another actin nucleator, persisted at the leading edge of the cells (Supplementary Fig. 13).

### Ser177 phosphorylation of Pdlim5 decreased Rac1 activity

**Figure 7:**
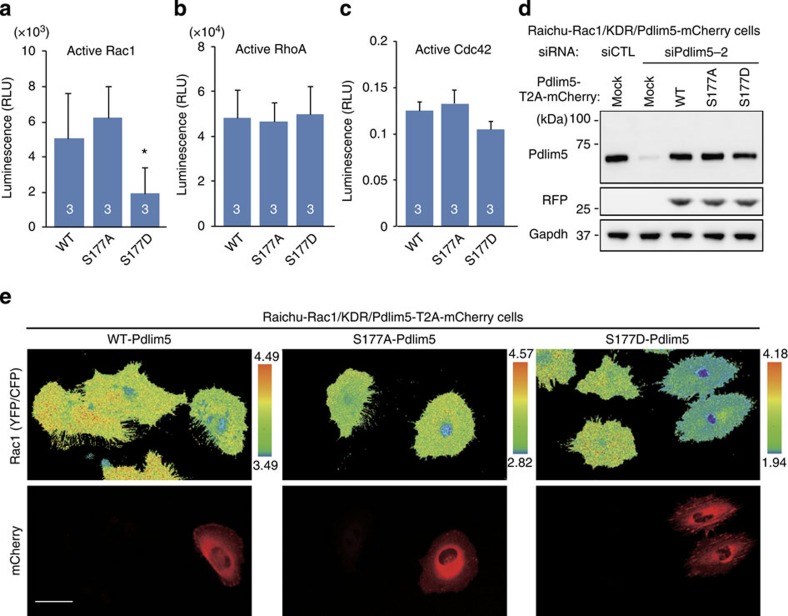
Ser177 phosphorylation of Pdlim5 suppressed Rac1 activity. (**a**–**c**) Activities of Rac1 (**a**), RhoA (**b**) and Cdc42 (**c**) in KDR cells were quantitated using G-LISA specific for Rac1, RhoA and Cdc42, respectively. Numbers in the bars indicate *n*. Data are representative of means±s.e.m. from three independent experiments. Significance of differences between series of results was assessed using one-way analysis of variance, followed by a *post-hoc* comparison with Dunnett’s method for multiple comparisons. *P<0.01 compared with KDR/EGFP-WT-Pdlim5. (**d**) The KDR system was established in vSMCs stably expressing FRET probes specific for Rac1 (Raichu-Rac1/vSMCs). Raichu-Rac1/vSMCs were transfected with either siCTL or siPdlim5-2. siPdlim5-2-resistant Pdlim5-T2A-mCherry (WT, S177A and S177D) was introduced via adenoviral-mediated gene delivery (Raichu-Rac1/KDR-Pdlim5-T2A-mCherry cells). TCLs were subjected to immunoblotting. (**e**) Imaging of Rac1 activity. Each type of Raichu-Rac1/KDR-Pdlim5-T2A-mCherry cell (WT, S177A and S177D) was imaged for YFP and CFP. FRET efficiencies are shown as YFP/CFP ratio images. Scale bars, 20 μm.

Next, we measured the activities of Rac1, an upstream regulator of the Arp2/3 complex that is required for lamellipodia formation^8^,^9^. GTP-bound active Rac1 was significantly reduced in KDR/S177D-Pdlim5 cells relative to KDR/WT- and KDR/S177A-Pdlim5 cells (Fig. 7a). Furthermore, we carried out imaging of Rac1 activity in living cells, using vSMCs stably expressing the Raichu-Rac1 probe (vSMC-R_Rac1_ cells), which is based on the principle of fluorescence resonance energy transfer (FRET) biosensors^21^. We established the KDR system in vSMC-R_Rac1_ cells, as described above, except that siPDLIM-resistant Pdlim5 was co-expressed with mCherry instead of being tagged with EGFP (Raichu-Rac1/KDR/Pdlim5-T2A-mCherry) (Fig. 7d). Rac1 activity was lower in cells expressing S177D-Pdlim5 than in those expressing WT-Pdlim5 or S177A-Pdlim5, especially in the cell periphery (Fig. 7e and Supplementary Fig. 14). Thus, these findings indicated that Rac1 activity was suppressed in cells expressing S177D-Pdlim5, especially in the cell periphery, resulting in dislocation of the Arp2/3 complex. On the other hand, we observed no changes in RhoA or Cdc42 activity (Fig. 7b,c and Supplementary Fig. 15).

### Pdlim5 phosphorylation altered the interaction with Arhgef6

**Figure 8:**
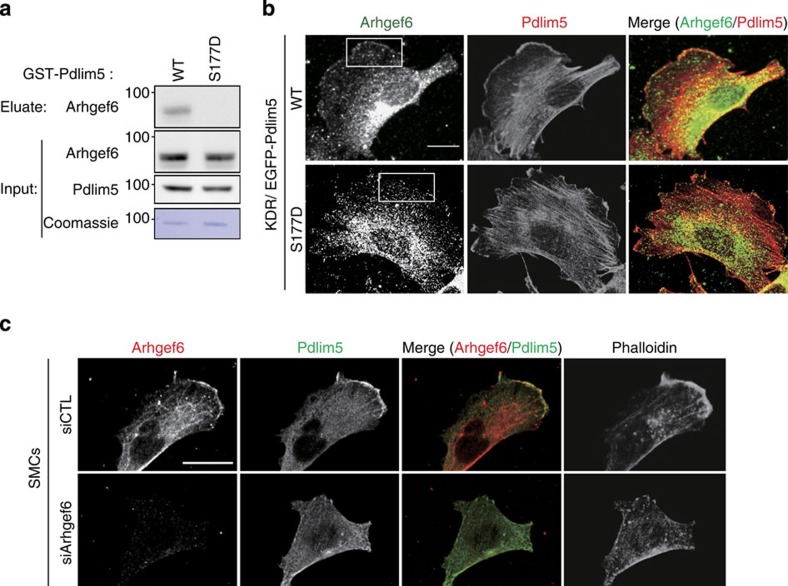
Ser177 phosphorylation of Pdlim5 disrupts its association with Arhgef6 at the cell periphery. (**a**) Immunoblotting analysis of GST-Pdlim5 pull-down assay. Eluates were subjected to immunoblotting with anti-Arhgef6 antibody. Coomassie staining demonstrates equal loading of GST-Pdlim5 proteins. (**b**) Immunostaining images of Arhgef6 and Pdlim5 from KDR/EGFP-Pdlim5 cells. Boxed area in KDR/EGFP-WT-Pdlim5 cell highlights representative co-localization of Arhgef6 with Pdlim5 at the cell periphery. Scale bars, 10 μm. (**c**) Immunostained images of Arhgef6 knockdown vSMCs stained with anti-Pdlim5 antibody, anti-Arhgef6 antibody and phalloidin. Scale bars, 10 μm.

To investigate the molecular mechanism by which S177D-Pdlim5 suppresses Rac1 activity, we performed GST pull-down assays followed by high-sensitivity shotgun liquid chromatography–mass spectrometry, using total cell lysates from U937 cells. Among a total of 1,225 proteins detected, several RhoGEFs were more prominently associated with GST-WT-Pdlim5 than with GST-S177D-Pdlim5 (Supplementary Table 1). Among them, we focused on Arhgef6, because it exclusively associated with WT-Pdlim5 (Fig. 8a) and can activate Rac1 at the leading edge of migrating cells^22^,^23^. Both the biochemical dissociation of Arhgef6 and S177D-Pdlim5, and the intracellular co-localization of Arhgef6 with Pdlim5 at the cell periphery were disrupted in KDR/S177D-Pdlim5 cells (Fig. 8b). These findings suggested that Ser177 phosphorylation of Pdlim5 disrupted the recruitment of Arhgef6 at the cell’s leading edge, potentially suppressing Rac1 activity. Next, we compared the effect of Arhgef6 knockdown on cell migration and morphology with that of Ser177 phosphorylation of Pdlim5 (Fig. 8c and Supplementary Fig. 16). The similarity was only partial: Arhgef6-knockdown vSMCs exhibited a disturbed migration relative to control vSMCs (Supplementary Fig. 16b–d) and defective lamellipodia (Fig. 8c), but not elevated formation of stress fibres (Fig. 8c).

### Pdlim5 recruits AMPK onto actin filaments

**Figure 9:**
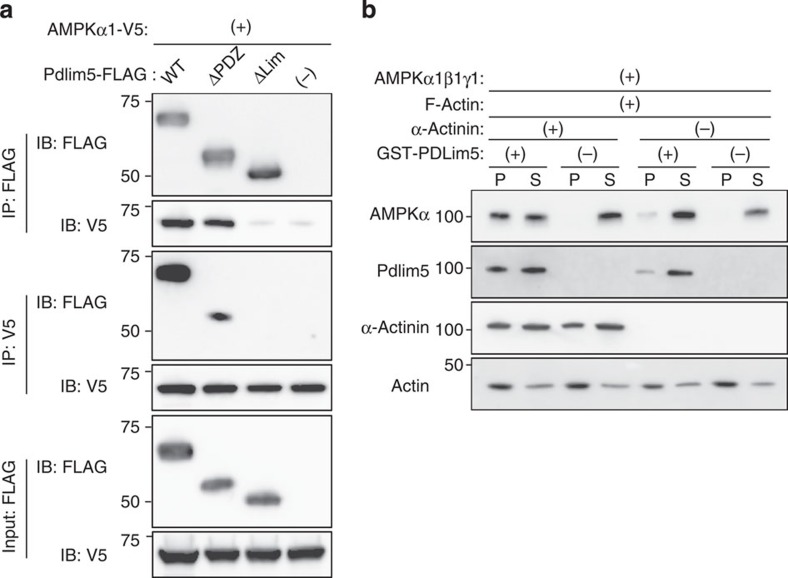
AMPK is recruited onto F-actin by directly binding the LIM domain of Pdlim5. (**a**) HEK293T cells were co-transfected with V5-tagged AMPKα and FLAG-tagged Pdlim5 (WT, ΔPDZ or ΔLIM domain). TCLs were immunoprecipitated by FLAG or V5 and then immunoblotted with the indicated antibodies. (**b**) F-actin binding assay of AMPK. AMPK was mixed with a fixed amount of F-actin in the presence or absence of α-actinin and GST-Pdlim5, incubated for 1 h at 24 °C and then centrifuged at 150,000 *g* for 1.5 h at 24 °C, to pellet the F-actin polymer and associated proteins. A sample of the pellet (P) and supernatant (S) were analysed by immunoblotting for the indicated antibodies.

To investigate how AMPK signalling is transmitted to peripheral actin filaments, we examined the physical link between actin filaments, Pdlim5 and AMPK. Immunoprecipitation/immunoblotting of HEK293T cells co-transfected with V5-tagged AMPKα and FLAG-tagged Pdlim5 (WT, ΔPDZ or ΔLIM) demonstrated that AMPK bound to Pdlim5 through the LIM domain (Fig. 9a). Next, we performed an F-actin-binding assay, in which F-actin and its binding proteins are found in the pellet fraction, to determine whether Pdlim5 promotes the recruitment of AMPK onto actin filaments. In the absence of Pdlim5, AMPK was found exclusively in the supernatant (Fig. 9b). However, in the presence of Pdlim5, AMPK shifted from the supernatant to the pellets and this shift was greatly stimulated by the presence of α-actinin (Fig. 9b). These findings indicate that Pdlim5 binds AMPK directly and promotes the recruitment of AMPK onto F-actin, a process mediated by α-actinin.

## Discussion

The results described here reveal the mechanism by which augmented AMPK activity inhibits cell migration. In this study, we serendipitously identified Pdlim5 as a novel substrate of AMPK. On augmentation of AMPK activity, Pdlim5 was phosphorylated at Ser177, disrupting its association with Arhgef6 at the cell periphery and suppressing Rac1 activity at the leading edge of cell. Suppression of Rac1 activity dislocated the Arp2/3 complex from the leading edge, resulting in attenuation of lamellipodia formation and inhibition of cell migration.

We previously established a unique screening method using two-step column chromatography combined with an *in vitro* kinase reaction. Using this method, we identified CLIP-170 as a novel AMPK substrate^4^. In this study, we identified Pdlim5 as another substrate of AMPK by probing cells with a pACC antibody after AMPK activation. The amino-acid sequence surrounding Ser177 of Pdlim5 is very similar to a sequence in ACC, which may explain why we were able to discover this new AMPK substrate using a pACC antibody. Importantly, Pdlim5 is not phosphorylated at all before stimulation with AMPK activators, whereas other AMPK substrates such as ACC and CLIP-170 (ref. 4) are already phosphorylated to some extent even before stimulation. These findings suggest that Pdlim5 is phosphorylated only under stressed conditions in which AMPK activity is augmented. Accordingly, we conclude that the functional impact of Pdlim5 phosphorylation by AMPK is exerted only under cellular conditions associated with augmented AMPK activity. Thus, Pdlim5 phosphorylation might cause different biological effects than phosphorylation of other AMPK substrates.

In mouse, the double knockout of AMPKα1α2 (AMPKα1^−/−^α2^−/−^) is early embryonic lethal^24^, but AMPKα-null MEFs are viable^25^. AMPK-null cells exhibit a metabolic shift towards aerobic glycolysis^26^, as well as abnormalities in cell polarity and cellular structures^2^. Pharmacological inhibition of AMPK also perturbs cell polarity and migration^4^. Thus, it is reasonable to conclude that AMPK activity affects directional cell migration by regulating cell polarity. However, as AMPK has many substrates other than Pdlim5, it is still difficult to infer the role of Pdlim5 phosphorylation from the lethal phenotype of the AMPKα1^−/−^α2^−/−^ mouse. On the other hand, two studies have reported different phenotypes of Pdlim5 homozygous knockout mice: dilated cardiomyopathy^27^ and embryonic lethality probably due to embryonic heart/circulation failure^28^. Pdlim5 associates with the actin cytoskeleton and promotes the assembly of protein complexes by acting as a scaffold protein, and thus recruit proteins that modulate cell architecture, actin dynamics and signal transduction^19^ to the actin filaments. Therefore, both phenotypes of Pdlim5 homozygous knockout mice may be attributed to the loss of organized cytoskeletal structures due to progressive loss of protein complex components. Similarly, we postulate that Pdlim5 and its phosphorylation play an important role in regulation of cell morphology and migration, although no previous reports have described the cell migratory behaviour of Pdlim5-knockout cells.

**Figure 10:**
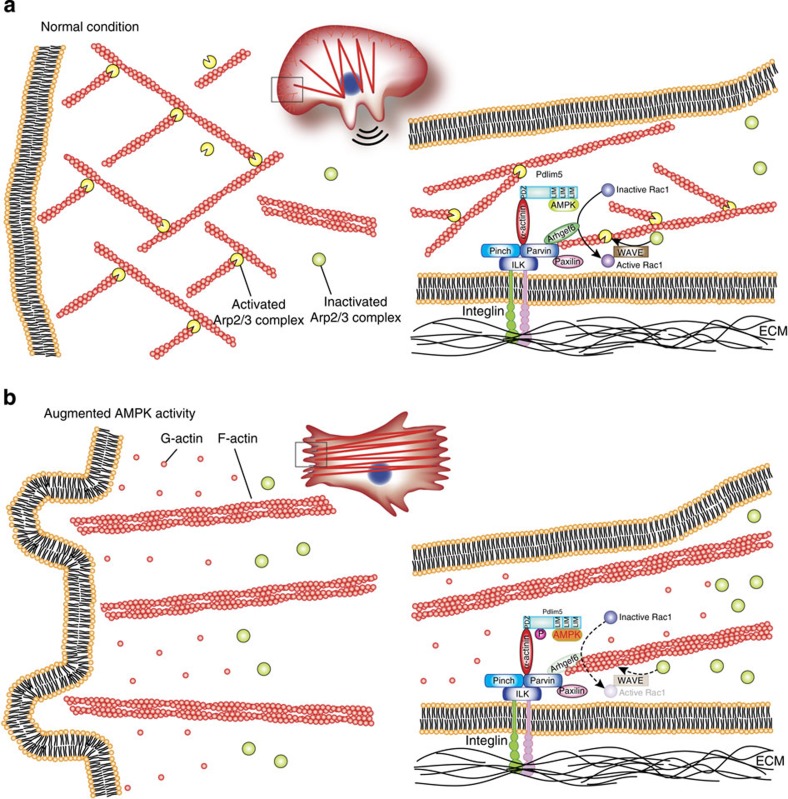
Model depicting how the Ser177 phosphorylation of Pdlim5 attenuates lamellipodia formation. (**a**) Under normal conditions, Arhgef6 recruited to the IPP complex at the cell periphery activates Rac1, contributing to efficient lamellipodia formation. (**b**) Once AMPK activity is augmented, Ser177 of Pdlim5 is phosphorylated, displacing Arhgef6 from the IPP complex and causing attenuation of lamellipodia. Boxed areas at the cell periphery are expanded, representing views from the top (left) and from the side (right); see text for explanation. ILK, integrin-linked kinase; PINCH, particularly interesting new cysteine-histidine-rich protein; Pdlim5, PDZ and LIM domain 5; WAVE, Wiskott–Aldrich Syndrome protein family verprolin homologous.

In this study, we focused on determining how AMPK-induced Ser177 phosphorylation of Pdlim5 inhibits cell migration. We found that Pdlim5 localized on actin-filament structures such as stress fibres and focal adhesions, as well as at the cell cortex (including lamellipodia), and that Ser177 phosphorylation itself did not influence Pdlim5 localization. However, the phosphomimetic mutant S177D-Pdlim5 caused characteristic morphological changes such as defective lamellipodia, enhanced ventral stress fibres and displacement of focal adhesions to the cell edge. These specific morphological changes at the cell edge were also observed on pharmacological activation of endogenous AMPK. Thus, the major initial morphological changes resulting from Ser177 phosphorylation of Pdlim5 seemed to arise from the cell cortex. Arhgef6 belongs to the Dbl family of GEFs, defined by the presence of tandem Dbl homology and Pleckstrin homology domains, and functions as a Rac-specific GEF at the cell periphery^22^,^23^,^29^. Arhgef6 is recruited to a signalling complex consisting of integrin-linked kinase, particularly interesting cysteine–histidine-rich protein and parvin (IPP complex) by binding to parvin^30^,^31^ (Fig. 10), which plays an important role in cell spreading and motility^32^, and may be involved in the Pdlim5 phosphorylation signal mediated by Arhgef6. The IPP complex assembles at small focal complexes at the tips of lamellipodia of migrating cells and then interacts with the cytoplasmic tails of β-integrin molecules to connect them to the actin cytoskeleton^32^. Thus, Arhgef6 activates Rac1 and reorganizes the actin cytoskeleton around the focal complex of lamellipodia, which is necessary for cell spreading and migration^29^. Moreover, α-actinin also binds to parvin directly^33^ and is involved in the IPP complex^31^. As Pdlim5 binds to α-actinin directly^34^, it is likely to be that Pdlim5 is recruited to the IPP complex in close proximity to Arhgef6 via binding to α-actinin. It is particularly noteworthy that S177D-Pdlim5 disrupted the physical association between Pdlim5 and displaced Arhgef6 from the cell periphery (Fig. 8). These findings led us to speculate that Arhgef6 is displaced from the IPP complex on Ser177 phosphorylation of Pdlim5 by AMPK, resulting in the suppression of Rac1 activity at the cell’s leading edge. Rac1 is predominantly localized at the plasma membrane^35^ and GTP-bound active Rac1 at the cell periphery can activate Arp2/3 complex through recruitment of the Wiskott–Aldrich Syndrome protein family verprolin homologous complex^10^,^11^. Therefore, we expected that the function of Arp2/3 complex would also be suppressed in KDR/S177D-Pdlim5 cells. In fact, the Arp2/3 complex was displaced from the cell periphery and distributed throughout the cytoplasm in KDR/S177D-Pdlim5 cells, consistent with a previous study demonstrating that inhibition of Rac1 activity interferes with intracellular localization of the Arp2/3 complex^36^. Furthermore, the morphological characteristics observed in KDR/S177D-Pdlim5 cells were quite similar to those in cells with a functional defect in the Arp2/3 complex^37^,^38^,^39^. As Arp2/3 complexes play a pivotal role in organizing branched actin filament networks to form lamellipodia^10^,^11^, the attenuation of lamellipodia formation and inhibition of cell migration observed in KDR/S177D-Pdlim5 cells was plausible. Thus, our findings strongly suggest that Ser177 phosphorylation of Pdlim5 by AMPK suppresses Rac1 activity by displacing Afhgef6 from the cell periphery, leading to functional suppression of the Arp2/3 complex. However, as the morphological phenotype of Arhgef6-knockdown cells was not entirely consistent with that of KDR/S177D-Pdlim5 cells, we must consider the idea that mechanisms other than the Arhgef6–Rac1–Arp2/3 complex pathway contribute to the phenotypes resulting from Ser177 phosphorylation of Pdlim5. One possible mechanism involves a relative increase in RhoA activity over Rac1 due to a reduction in Rac1 activity, which may contribute to the phenotype even in the absence of elevated RhoA activity. Consistent with this idea, the relative balance between Rac1 and RhoA activities regulates cell morphology and migratory behaviour^40^,^41^. Another possibility is that other GEFs or GTPase-activating proteins contribute to the phenotype of KDR/S177D-Pdlim5 cells. Indeed, dedicator of cytokinesis 2 is also a Rac-specific GEF and interacted with WT-Pdlim5 more strongly than with S177D-Pdlim5 (Supplementary Table 1).

Another important question relates to the mechanism underlying excessive formation of stress fibres, observed in both vSMCs treated with AMPK activator and KDR/S177D-Pdlim5 cells. In particular, cells stimulated with AMPK activators exhibited striking elongation of dorsal stress fibres from the cell periphery. This finding was in excellent agreement with a previous report demonstrating that Arp2/3-defective cells exhibited a higher growth rate of dorsal stress fibres^39^. The authors of that report proposed that the elevated growth of dorsal stress fibres may result from the increased concentration of cytoplasmic G-actin caused by the absence of Arp2/3-nucleated barbed ends^10^. In addition, mDia remained localized at the cell periphery in KDR/S177D-Pdlim5 cells, whereas Arp2/3 complex moved from the periphery to the cytosol. mDia is another major actin filament nucleator that nucleates linear actin filament at the cell periphery and can elongate actin directly proportional to the G-actin monomer concentration^42^. Thus, mDia at the cell periphery under increased G-actin concentration may also contribute to elevated formation of stress fibres and filopodia in KDR/S177D-Pdlim5 cells. Taken together, these data indicate that the morphological and migratory phenotypes observed in cells expressing Ser177-phosphorylated Pdlim5 might be caused not only by the Arhgef6–Rac1–Arp2/3 complex pathway, but also by other additional mechanisms/pathways including other GEFs/GTPase-activating proteins, other Rho-GTPases (RhoA and Cdc42) and mDia. In addition, the data in Supplementary Fig. 6 demonstrate that KDR/S177A-Pdlim5 cells moved more slowly when treated with AMPK activator, indicating that Pdlim5 is the primary regulator of migration downstream of AMPK, although other pathways are also involved.

We observed a physical and functional association between AMPK, Pdlim5 and F-actin. Pdlim5 binds to AMPK directly through its LIM domain and to F-actin through its PDZ domain, which places AMPK close to F-actin. This interaction seems to rapidly and efficiently transmit AMPK signalling to the actin filament architecture. Thus, we propose that once energy depletion occurs and AMPK is activated, the signal might be transmitted to peripheral actin filaments through this complex, leading to the remodelling of actin-filament architecture and inhibition of cell migration.

A striking feature of the regulation of cell migration by AMPK activity level is that both suppression and augmentation of AMPK inhibit cell migration. Cell migration is a highly complex behaviour that is accomplished by tightly orchestrated dynamic remodelling of the actin cytoskeleton and microtubule network^16^. We previously reported that suppression of AMPK activity inhibits cell migration by hyperstabilizing the microtubule cytoskeleton via dephosphorylation of the microtubule plus-end protein CLIP-170 (ref. 4). In this study, we demonstrated that augmentation of AMPK activity inhibits cell migration by reorganizing actin filaments through phosphorylation of Pdlim5. Thus, the effects of high or low AMPK activity on cell migration may be mediated by completely different mechanisms acting on different substrates, and the two types of substrates may regulate cell migration separately by sensing the level of AMPK activity between two extremes.

## Methods

### Reagents and antibodies

The following reagents were purchased from the indicated suppliers: AICAR (Sigma-Aldrich), 2-DG (Sigma-Aldrich), A769662 (Santa Cruz Biotechnology) and GST-AMPKα1/β1/γ1 (Carna Biosciences). The following antibodies were purchased from the indicated suppliers: anti-AMPKα (1:2,000; Cell Signaling, 2603), phospho-Thr172 AMPKα (1:2,000; Cell Signaling, 2535), ACC (1:2,000; Cell Signaling, 3676), phospho-Ser79 ACC (1:2,000; Cell Signaling, 3661), anti-paxillin (1:1,000, Zymed Laboratories), anti-α-actinin (D6F6) (1:1,000 for immunoblot; Cell Signaling, 6487), monoclonal anti-α-actinin (1:1,000 for immunostain; Sigma-Aldrich, A5044), anti-Arp2 antibody (1:1,000; Cell Signaling, 3128), anti-Arp3 (FMS338) (1:500 for immunostaining; Abcam, ab49671), anti-Arpc2 (EPR8533) (1:2,000; Abcam, ab133315), anti-Arhgef6/Cool2/αPIX (C23D2) (1:1,000 for immunoblot; 1:400 for immunostain; Cell Signaling, 4573), anti-Gapdh antibody (1:5,000; Millipore, MAB374), anti-GFP-horseradish peroxidase (HRP) (1:3,000; MBL, 598-7), anti–RFP-HRP (1:3,000; MBL, PM005-7), anti-FLAG M2-HRP (1:5,000; Sigma-Aldrich, A8592), anti-V5-HRP antibody (1:5,000; Life Technologies, R961-25), HRP-coupled goat anti–rabbit (1:8,000; Cappel, 55696), anti-mouse IgG (1:8,000; Cappel, 55550), Alexa Fluor 488- (1:1,000 for staining; Life Technologies, A11029), Alexa Fluor 546- (1:1,000 for staining; Life Technologies, A11003) and Alexa Fluor 568-labelled secondary antibodies (1:1,000 for staining; Life Technologies, A11011), and Alexa Fluor 647 phalloidin (1:100 for staining; Cell Signaling, 8940). Anti-FLAG M2 affinity gel (A2220) and anti-V5 agarose affinity gel (A7345) were from Sigma-Aldrich. We used three different AMPK activators: AICAR is metabolized intracellularly to ZMP (5-aminoimidazole-4-carboxamide-1-β-D-ribofuranotide), an AMP analogue. A769662 is a thienopyridone derivative that acts as an allosteric activator of the AMPK by binding to an alternative site that does not overlap with the AMP-binding site. 2-DG is a non-metabolizable glucose analogue and inhibitor of phosphohexose isomerase that inhibits glycolysis and mimics glucose starvation, increases the AMP/ATP ratio and thereby activates AMPK.

*Antibodies for Pdlim5 and pS177 of Pdlim5*. Six polyclonal Pdlim5 antibodies (1:1,000 for immunoblotting and immunostaining) and two polyclonal phospho-Ser177 (pS177)-Pdlim5 antibodies (1:1,000 for immunoblotting) were generated as follows. Three different peptides corresponding to mouse Pdlim5 sequences (amino acids 229–245, QGDIKQQNGPPRKHIVEC; amino acids 290–306, CTGTEHLTESENDNTKKA; and amino acids 381–397, SSGTGASVGPPQPSDQDC), as well as Ser-phosphorylated and non-phosphorylated peptides corresponding to the mouse Pdlim5 sequences surrounding Ser177 (amino acids 172–182, LHLSA(pS)GLHVS), were chemically synthesized. Rabbits were immunized five times with the keyhole limpet haemocyanin–phosphopeptide conjugates mixed with Freund’s complete adjuvant and bled 7 days after the last immunization. Phosphopeptide-reactive antibody was captured by a column containing phosphopeptide-conjugated Sepharose. The antibodies were then eluted, and those reactive to sequences other than phosphoserine were removed using a column containing non-phosphorylated peptides. Specific reactivity with the targeted phosphoserine sequence was confirmed by ELISA using phosphorylated and non-phosphorylated peptides.

*Cell culture and siRNA transfection.* C2C12 cells (an immortalized mouse myoblast cell line) and HEK293T cells were obtained from the American Type Culture Collection. A vSMC line established from thoracic aorta of a p53-knockout mice (P53LMACO1) was purchased from Health Science Research Resources Bank. These cells were maintained in DMEM medium (Sigma-Aldrich) supplemented with 10% FCS (Equitech-Bio) and 1% penicillin–streptomycin at 37 °C in a 5% CO_2_ atmosphere at constant humidity and passaged by trypsinization at 70–80% confluence. HL60 cells and RAW264.7 cells were obtained from the Japanese Collection of Research Bioresources Cell Bank and American Type Culture Collection, respectively. These cells were maintained in RPMI1640 medium with 10% FCS and 1% penicillin–streptomycin. HEK293T cells and vSMCs were transfected with plasmids using Lipofectamine 2000 reagent (Invitrogen). To knock down endogenous Pdlim5, C2C12 cells and vSMCs were transfected with siRNAs (30 nM) targeting Pdlim5 (siPdlim5-1, sense: 5′-ggaacaauaugucguggauTT-3′; antisense: 5′-auccacgacauauuguuccTT-3′; siPdlim5-2, sense: 5′-ggguaguagcuaugagaauTT-3′; antisense: 5′-auucucauagcuacuacccTT-3′) using Lipofectamine RNAiMAX (Invitrogen). To knock down endogenous Arpc2, vSMCs were transfected with siRNAs (5 nM) targeting Arpc2 (siArpc2-1, sense: 5′-ggccuauauucauacacgaTT-3′; antisense: 5′-ucguguaugaauauaggccTT-3′; siArpc2-2, sense: 5′-gaaccaggauauaauguuuTT-3′; antisense: 5′-aaacauuauauccugguucTG-3′) using Lipofectamine RNAiMAX (Invitrogen). RAW264.7 cells (mouse leukemic monocyte macrophage cell) were used to check the effect of siRNAs against Arhgef6. RAW264.7 cells were transfected with siRNAs (50 nM) targeting Arhgef6 (siArhgef6-1, sense: 5′-gauucuuaaggugaucgaaTT-3′; antisense: 5′-uucgaucaccuuaagaaucTG-3′; siArhgef6-2, sense: 5′-gugaugaucuagaacgauuTT-3′; antisense: 5′-aaucguucuagaucaucacTG-3′) using GenMute siRNA Transfection Reagent for RAW 264.7 (SignaGen). siControl was used as a negative control. Efficiency of siRNA-mediated knockdown was confirmed at 24 and 72 h after incubation with siRNAs. AMPKα double-knockout (AMPKα1^−/−^α2^−/−^) MEFs (AMPKα-null MEFs)^25^ were kindly provided by Dr B. Viollet (INSERM, France) and maintained in DMEM supplemented with 10% FCS, 1 mM sodium pyruvate and 1% penicillin–streptomycin at 37 °C in a 5% CO_2_ atmosphere at constant humidity.

### Hypoxia

Cultured cells were exposed to hypoxia for 2 h. Hypoxic conditions (1% O_2_) were maintained in a MCO-5M multi-gas incubator (Sanyo).

### Establishment of the KDR system

To replace endogenous Pdlim5 by EGFP-tagged recombinant Pdlim5 (WT, S177A or S177D), vSMCs were transfected with siPdlim5-2 to deplete endogenous Pdlim5. Next, 12 h after siPdlim5-2 transfection, siPdlim5-2-resistant EGFP-tagged Pdlim5 (WT, S177A or S177D) was adenovirally transduced into vSMCs, to establish the KDR system. vSMCs were incubated with adenovirus at a multiplicity of infection of 10 in DMEM supplemented with 10% FCS at 37 °C under 5% CO_2_ for 30 min, with gentle mixing every 10 min, and then further incubated for 48 h before analysis.

### Purification and identification of Pdlim5

C2C12 cells seeded on 15-cm dishes (2 × 10^6^ cells per dish) were treated with 2 mM of AICAR for 1 h, harvested and lysed on ice in lysis buffer A (20 mM Tris pH 8.0, 0.5% NP-40, 0.5% CHAPS, 20% acetonitrile, 25 mM β-glycerophosphate, 10 mM NaF and protease inhibitor cocktail (Nacalai Tesque)). Lysates were incubated at 4 °C with agitation for 20 min, followed by centrifugation at 10,000 *g* for 20 min. Supernatant from six 15-cm dishes was passed through a 0.45-μm sterilization filter and loaded onto a TSK-GEL SuperQ-5PW (7.5 × 75 mm, TOSOH) anion-exchange column pre-equilibrated with column buffer A (20 mM Tris pH 8.0, 0.5% CHAPS, 20% acetonitrile, 25 mM β-glycerophosphate, 10 mM NaF). After being washed with column buffer A, proteins were eluted with a linear gradient of NaCl (0–1 M over 60 min) at a flow rate of 0.5 ml min^−1^. Fractions (0.5 ml each) were collected and a 50-μl aliquot of each fraction was analysed by immunoblotting with anti-pACC antibody. The corresponding fractions were prepared in the presence of 0.3% TFA (trifluoroacetic acid), 0.1% OG (*n*-octyl-β-D-thioglucopyranoside) and 20% acetonitrile, and loaded onto a Protein-R (4.6 × 250 mm, Nacalai Tesque) reverse-phase HPLC column pre-equilibrated with column buffer B (0.1% TFA and 0.1% OG). After being washed with column buffer B, the proteins were eluted with a linear gradient of acetonitrile (20–80% over 60 min) at a flow rate of 0.5 ml min^−1^. Fractions (0.5 ml each) were collected and a 25-μl aliquot of each fraction was analysed by immunoblotting with anti-pACC antibody. The corresponding fractions were again prepared in the presence of 0.3% TFA, 0.1% OG and 20% acetonitrile, and loaded onto a 5Ph-AR-300 (4.6 × 250 mm, Nacalai Tesque) reverse-phase HPLC column pre-equilibrated with column buffer B. After being washed with column buffer B, proteins were eluted with a linear gradient of acetonitrile (20–80% over 60 min) at a flow rate of 0.5 ml min^−1^. Each fraction was analysed by SDS–PAGE and visualized by silver staining and immunoblotting with anti-pACC antibody. Target bands matching the pACC antibody cross-reacting bands were excised from the gel and analysed using matrix-assisted laser desorption/ionization-quadrupole-time-of-flight-tandem mass spectrometry (MALDI-Qq-TOF MS/MS).

### Protein purification

Recombinant FLAG-tagged Pdlim5 proteins were purified as follows: HEK293T cells transfected with pEF-DEST51/cFLAG plasmid encoding WT Pdlim5, S175A Pdlim5 or S177A Pdlim5 were lysed in lysis buffer B (20 mM MOPS pH 7.5, 0.15 M NaCl, 0.5% CHAPS, 1 mM EDTA, 1 mM dithiothreitol (DTT) and protease inhibitor cocktail) and immunoprecipitated with anti-FLAG M2 agarose (Sigma-Aldrich) at 4 °C for 30 min. The beads were washed three times with wash buffer (20 mM MOPS pH 7.5, 0.3 M NaCl, 0.5% CHAPS, 1 mM EDTA, 1 mM DTT and protease inhibitor cocktail) and eluted with elution buffer (20 mM MOPS pH 7.5, 0.3 M NaCl, 0.5% CHAPS, 1 mM DTT and 0.5 mg ml^−1^ FLAG peptide (Sigma-Aldrich)) at 4 °C for 30 min. After centrifugation, the supernatants were used as recombinant FLAG-tagged proteins. Recombinant GST-Pdlim5 proteins (WT, S177A, S177D and ΔPDZ) were purified as follows: BL21 chemically competent *E. coli* (Invitrogen) were transformed with pGEX-6P-1-WT Pdlim5, pGEX-6P-1-S177A Pdlim5, pGEX-6P-1-S177D Pdlim5 or pGEX-6P-1-ΔPDZ Pdlim5, and then induced with 0.5 mM isopropyl-β-D-thiogalactoside (Sigma-Aldrich) at 25 °C for 10 h. The cells were collected by centrifugation and lysed by sonication in PBS containing 5 mM EDTA and protease inhibitor cocktail. After addition of 1% Triton X-100, the lysates were agitated at 4 °C for 30 min and pulled down with glutathione–Sepharose 4 Fast Flow (GE Healthcare) at 4 °C for 1 h. After being washed three times, the proteins were eluted with 15 mM reduced glutathione and ultrafiltered in elution buffer using a Nanosep 10K Device (Pall Life Science).

### Immunoblotting

Immunoblotting was performed using the indicated antibodies. Blots were cropped such that at least one marker position is present. Uncropped full scans of the figures are supplied in Supplementary Figs 17–23.

### Phosphorylation assay

Phosphorylation assays were carried out at 30 °C in a reaction volume of 10 μl containing Tris-HCl (20 mM pH 7.4), glycerol (10%), NaCl (0.3 mM), AMP (0.2 mM), MgCl2 (10 mM), γ-^32^P ATP (GE Healthcare BioScience, 10 μCi, 1.7 pmol) and AMPK purified from rat liver (2 ng μl^−1^). Purified mouse Pdlim5-cFLAG proteins or purified recombinant GST-fused mouse Pdlim5 proteins were used as substrates. After 60 min, reactions were terminated and ultrafiltered in PBS containing 0.1% SDS using a Nanosep 10K Device (Pall Life Science). Each fraction sample was analysed by SDS–PAGE and visualized by autoradiography. For immunoblotting with anti-pS177 antibody, recombinant GST-fused Pdlim5 proteins were incubated under the same conditions, except that ATP (0.1 mM) was included instead of γ-^32^P ATP.

### Scratch assay

KDR/EGFP-Pdlim5 (WT, S177A and S177D) cells or MEFs were plated on a collagen-coated 35-mm glass dish at a density of 5 × 10^5^ cm^−2^. Eight hours after plating, a scratch was made with a P-200 pipette tip and the lesions were observed for a total of 8 h. Differential interference contrast images were recorded every 5 min using an Olympus LCV110 incubator microscope (Olympus Corporation, Tokyo, Japan). To determine cell trajectories, the centrioles of cell nuclei were tracked throughout time-lapse movies, and migration-tracking images were generated using the MetaMorph 7.1.3.0 software (MDS Analytical Technologies, Downingtown, PA, USA). Overall migration speed was calculated as the average of migration speeds measured every 5 min.

### Single-cell migration assay

KDR/EGFP-Pdlim5 (WT, S177A and S177D) cells were plated on a collagen-coated 35-mm glass dish at a density of 5 × 10^5^ cm^−2^. Five hours after the plating, we started to observe cell migration by recording differential interference contrast images every 5 min for a total of 4 h using an Olympus LCV110 incubator microscope (Olympus Corporation).

### Immunocytochemistry and fluorescence imaging

vSMCs or KDR cells were seeded on a collagen-coated 35-mm glass dishes (Asahi Techno Glass Corporation, Chiba, Japan). After cells firmly attached to the dish, they were washed once with warm PBS and fixed with 4% paraformaldehyde for 5 min at room temperature. Next, the cells were permeabilized with 0.1% Triton X-100 in PBS for 5 min at room temperature and then blocked with 1% BSA at 4 °C overnight. The next day, samples were immunostained with primary antibodies (1:1,000 in 1% BSA, 1 h). For secondary reactions, species-matched Alexa Fluor 488- or Alexa Fluor 568-labelled secondary antibody was used (1:1,000 in 1% BSA, 30 min). Just before imaging, the sample was incubated with Alexa Fluor 647-conjugated phalloidin in CGS-Sol A for 1 h. Fluorescence images of EGFP, Alexa Fluor 488, Alexa Fluor 546, Alexa Fluor 568 and Alexa Fluor 647 were recorded using an Olympus FV1000-D confocal laser scanning microscope (Olympus Corporation) equipped with a cooled charge-coupled device CoolSNAP-HQ camera (Roper Scientific, Tucson, AZ, USA) and a PLAPO × 60 oil-immersion objective lens. To measure the paxillin-positive area, all intensity profiles were analysed using the MetaMorph 7.1.3.0 software.

### Time-lapse imaging of KDR/EGFP-Pdlim5 cells

KDR/EGFP-Pdlim5 (WT and S177A) cells were seeded on collagen-coated 35-mm glass dishes at a density of 4 × 10^4^ cm^−2^. Five hours after plating, cells were treated with AICAR (2 mM). The fluorescence images were recorded from 10 min before to 60 min after AICAR treatment, using an Olympus IX-81 inverted fluorescence microscope (Olympus Corporation) equipped with a cooled charge-coupled device CoolSNAP-HQ camera (Roper Scientific) and a PLAPO × 60 oil-immersion objective lens controlled by MetaMorph version 7.1.3.0. An EGFP image was obtained every 30 s through a U-MNIBA2 filter (Olympus Corporation), which had a 470–495 excitation filter and a 510–550 emission filter. Cells were maintained on a microscope at 37 °C with a 5% carbon dioxide mixture using a stage-top incubator (Tokai Hit). MetaMorph was used to convert a series of time-lapse images to video format.

### Measurement of the GTP-bound form of Rac1, RhoA and Cdc42

KDR cells at 50%confluence were incubated for 24 h in FCS-free DMEM to starve the cells, and then treated with 10% FCS for 30 min. Cell lysates were collected and levels of activated GTP-bound Rac1, RhoA and Cdc42 were determined using the G-LISA Rac1 Activation Assay Biochem Kit (BK126, Cytoskeleton Inc., CO, USA), G-LISA RhoA Activation Assay Biochem Kit (BK121, Cytoskeleton Inc.) and G-LISA Cdc42 Activation Assay Biochem Kit (BK127, Cytoskeleton Inc.), respectively.

### Imaging of Rho GTPases (Rac1, RhoA and Cdc42) activities

To visualize activities of Rho GTPases in living cells, we established a vSMC cell line stably expressing a Rho GTPase FRET biosensor consisting of cyan fluorescent protein (CFP) and yellow fluorescent protein (YFP), as previously described^43^. In brief, vSMCs were transfected with the CFP/YFP-type FRET biosensor gene for Rho GTPases, using the PiggyBac retrotransposon-mediated gene transfer system, and then cultured for 2 w with 10 μg ml^−1^ blasticidin S to select for vSMCs stably expressing the FRET biosensor (Raichu-Rac1, RhoA or Cdc42/vSMC). Next, a KDR system for vSMCs expressing Raichu-Rac1, Raichu-RhoA or Rhaicu-Cdc42 was established as described above, except for the use of an adenovirus encoding non-tagged Pdlim5 (WT, S177A and S177D)-T2A-mCherry instead of EGFP-tagged Pdlim5 (Raichu-Rac1, RhoA or Cdc42/KDR-Pdlim5-T2A-mCherry). Forty-eight hours after the transduction of Pdlim5-T2A-mCherry, Raichu-Rac1, RhoA or Cdc42/KDR-Pdlim5-T2A-mCherry cells were re-plated on collagen-coated glass-base dishes. All experiments were performed at 37 °C in a 5% CO_2_ atmosphere using a heating chamber. Dual images for CFP and YFP were obtained on a laser scanning microscope (LSM710, Zeiss) using an excitation wavelength of 405 nm and emission bandpass filters of 458–510 nm for CFP and 517–598 nm for YFP. After background subtraction, FRET/CFP ratio images were created using the LSM software ZEN2011 (Zeiss) and displayed as an intensity-modulated display image. For quantitative FRET analysis, the FRET/CFP ratio of each cell was calculated by dividing the fluorescence intensity of YFP by that of CFP over the total cellular area.

### GST pull-down assay

For identification of proteins associated with Pdlim5, 20 μg of purified GST-tagged Pdlim5 WT or S177D-Pdlim5 was immobilized on glutathione–Sepharose beads and incubated at 4 °C for 2 h with cell lysate from 2 × 10^9^ U937 cells in lysis buffer (30 mM MOPS pH 7.5, 150 mM NaCl, 0.5% Triton-X100, 1.5 mM MgCl_2_, 1 mM EGTA, 1 mM DTT and protease inhibitor cocktail). After three washes of the beads with lysis buffer, associated proteins were eluted in elution buffer (30 mM MOPS pH 7.5, 500 mM NaCl, 0.5% Triton-X100, 1.5 mM MgCl_2_, 1 mM EGTA, 1 mM DTT and protease inhibitor cocktail) and then subjected to silver staining and high-sensitivity shotgun liquid chromatography–mass spectrometry (LTQ Orbitrap ELITE, Thermo Scientific).

### F-actin binding assay of AMPK

F-actin binding assays were performed using the Actin Binding Protein Biochem Kit (BK001, Cytoskeleton, Inc.) according to the manufacturer’s protocol, with minor modifications. Briefly, 1 μl of F-actin (21 μM actin) in actin polymerization buffer (100 mM KCl, 2 mM MgCl_2_, 0.5 mM ATP, 0.2 mM Tris-HCl, pH 8.0) was mixed with 0.5 μl of purified AMPK (200 ng μl^−1^) in the presence or absence of GST-tagged WT-Pdlim5 and 1 μl of α-actinin (1 μg μl^−1^) in 60 μl reaction volume, incubated for 1 h at 24 °C and then centrifuged at 150,000 *g* for 1.5 h at 24 °C, to pellet the F-actin polymer and associated proteins. Equal amounts of pellet (P) and supernatant (S) were resolved by SDS–PAGE and analysed by immunoblotting with the indicated antibodies.

### Statistical analyses

Box plots show the entire population. Other data are expressed as means±s.e.m. Two-tailed Student’s *t*-test was used to analyse differences between two groups. Differences among multiple groups were compared by one-way analysis of variance, followed by a *post-hoc* comparison with Dunnett’s method using the JMP 8.0.1 software (SAS Institute Inc., Cary, NC, USA). *P*<0.01 was considered to indicate statistically significant differences.

## Authors contributions

Y.Y. and O.T. designed and conducted the study, performed most of the experiments and wrote the manuscript. S.T. designed and wrote the manuscript. A.N. discussed the results and helped to write the manuscript. H.K. and K.T. performed the proteomic analysis. H.K. conducted and supported the biological experiments. N.I. performed the biochemical experiments and helped to generate plasmids. S.H., S.Y., Y.S., K.M. and Y.A. discussed the results and reviewed the manuscript. Y.L. H.A., M.A. and T.M. discussed the results. M.K. supervised all work.

## Additional information

**How to cite this article:** Yan, Y. *et al.* Augmented AMPK activity inhibits cell migration by phosphorylating the novel substrate Pdlim5. *Nat. Commun.* 6:6137 doi: 10.1038/ncomms7137 (2015).

## Supplementary Material

Supplementary Information Supplementary Figures 1-23, Supplementary Table 1 and Supplementary Methods.

Supplementary Movie 1a Time-lapse images of scratch assay of KDR/EGFP-Pdlim5 cells depicted in Fig. 3d. (a) WT, (b) S177A, and (c) S177D. DIC images were obtained every 5 min for a total 8 h using an incubator microscope (LCV110; Olympus Corporation, Tokyo, Japan).

Supplementary Movie 1b Time-lapse images of scratch assay of KDR/EGFP-Pdlim5 cells depicted in Fig. 3d. (a) WT, (b) S177A, and (c) S177D. DIC images were obtained every 5 min for a total 8 h using an incubator microscope (LCV110; Olympus Corporation, Tokyo, Japan).

Supplementary Movie 1c Time-lapse images of scratch assay of KDR/EGFP-Pdlim5 cells depicted in Fig. 3d. (a) WT, (b) S177A, and (c) S177D. DIC images were obtained every 5 min for a total 8 h using an incubator microscope (LCV110; Olympus Corporation, Tokyo, Japan).

Supplementary Movie 2a Time-lapse images of single cell migration assay of KDR/EGFP-Pdlim5 cells depicted in Supplementary Fig. 7a. (a) WT, (b) S177A, and (c) S177D. DIC images were obtained every 5 min for a total 4 h using an incubator microscope (LCV110; Olympus Corporation, Tokyo, Japan).

Supplementary Movie 2b Time-lapse images of single cell migration assay of KDR/EGFP-Pdlim5 cells depicted in Supplementary Fig. 7a. (a) WT, (b) S177A, and (c) S177D. DIC images were obtained every 5 min for a total 4 h using an incubator microscope (LCV110; Olympus Corporation, Tokyo, Japan).

Supplementary Movie 2c Time-lapse images of single cell migration assay of KDR/EGFP-Pdlim5 cells depicted in Supplementary Fig. 7a. (a) WT, (b) S177A, and (c) S177D. DIC images were obtained every 5 min for a total 4 H using an incubator microscope (LCV110; Olympus Corporation, Tokyo, Japan).

Supplementary Movie 3a Time-lapse images of scratch assay of WT-MEFs and AMPK-null MEFs in the absence (a and c) or presence (b and d) of AICAR (1 mM) treatment depicted in Fig. 4b. DIC images were obtained every 5 min for a total 8 h using an incubator microscope (LCV110; Olympus Corporation, Tokyo, Japan).

Supplementary Movie 3b Time-lapse images of scratch assay of WT-MEFs and AMPK-null MEFs in the absence (a and c) or presence (b and d) of AICAR (1 mM) treatment depicted in Fig. 4b. DIC images were obtained every 5 min for a total 8 h using an incubator microscope (LCV110; Olympus Corporation, Tokyo, Japan).

Supplementary Movie 3c Time-lapse images of scratch assay of WT-MEFs and AMPK-null MEFs in the absence (a and c) or presence (b and d) of AICAR (1 mM) treatment depicted in Fig. 4b. DIC images were obtained every 5 min for a total 8 h using an incubator microscope (LCV110; Olympus Corporation, Tokyo, Japan).

Supplementary Movie 3d Time-lapse images of scratch assay of WT-MEFs and AMPK-null MEFs in the absence (a and c) or presence (b and d) of AICAR (1 mM) treatment depicted in Fig. 4b. DIC images were obtained every 5 min for a total 8 h using an incubator microscope (LCV110; Olympus Corporation, Tokyo, Japan).

Supplementary Movie 4a Time-lapse images of KDR/EGFP-WT-Pdlim5 cells (a) or KDR/EGFP-S177A-Pdlim5 cells (b) treated with AICAR (2 mM) depicted in Fig. 5b and 5c, respectively. EGFP images were obtained before and after the treatments for a total 60 min using an Olympus IX-81 inverted fluorescence microscope (Olympus Corporation) equipped with a cooled CCD CoolSNAP-HQ camera (Roper Scientific).

Supplementary Movie 4b Time-lapse images of KDR/EGFP-WT-Pdlim5 cells (a) or KDR/EGFP-S177A-Pdlim5 cells (b) treated with AICAR (2 mM) depicted in Fig. 5b and 5c, respectively. EGFP images were obtained before and after the treatments for a total 60 min using an Olympus IX-81 inverted fluorescence microscope (Olympus Corporation) equipped with a cooled CCD CoolSNAP-HQ camera (Roper Scientific).
