# Supplementary material for: Augmented AMPK activity inhibits cell migration by phosphorylating the novel substrate Pdlim5
Source: Nat Commun. 2015 Jan 30;6:6137. doi: 10.1038/ncomms7137 (PMC4317497; doi:10.1038/ncomms7137)
Supplement: Supplementary Information — Supplementary Figures 1-23, Supplementary Table 1 and Supplementary Methods. [file ncomms7137-s1.pdf]

Supplementary Figure 1

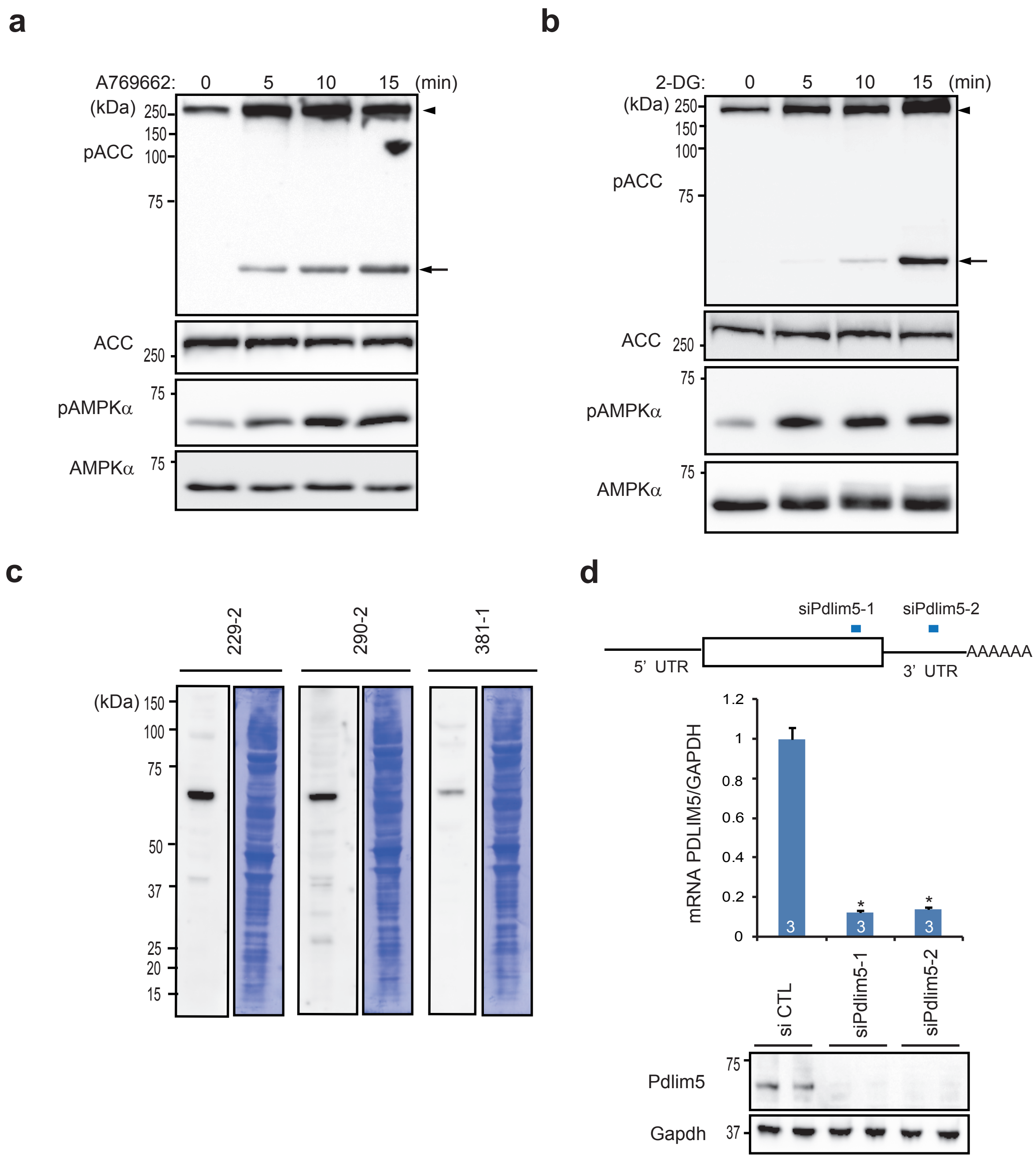

Supplementary Figure 1. Pdlim5 is a substrate of AMPK. (a and b) C2C12 cells were stimulated with either A-769662 (100 μM) (a) or 2-DG (25 mM) **(b)** 12 h after serum starvation. Total cell lysates (TCLs) were harvested at 0, 5, 10, or 15 min after stimulation and subjected to immunoblotting with anti-pACC antibody. Cross-reacting bands, p64 (arrows), were detected in a time-dependent manner only after treatment, in addition to the bands corresponding to pACC (arrowheads). **(c)** Validation of original polyclonal antibodies against mouse Pdlim5 (229-2, 290-2, and 381-1). TCLs from C2C12 cells were subjected to immunoblotting with these antibodies. **(d)** A schematic figure (upper panel) showing the positions of two different siRNAs targeting mouse Pdlim5 (siPdlim5-1 and siPdlim5-2). Quantitative RT-PCR (middle panel) and immunoblotting (lower panel) analysis of siRNA-treated C2C12 cells demonstrated that both siRNAs knocked down endogenous Pdlim5 with high efficiency. Numbers in the bars indicate *n*. Data are representative of means ± s.e.m from two independent experiments. Significance of differences between series of results was assessed using one-way ANOVA, followed by a post hoc comparison with Dunnett's method for multiple comparisons. *$P < 0.01$ relative to siCTL.

Supplementary Figure 2

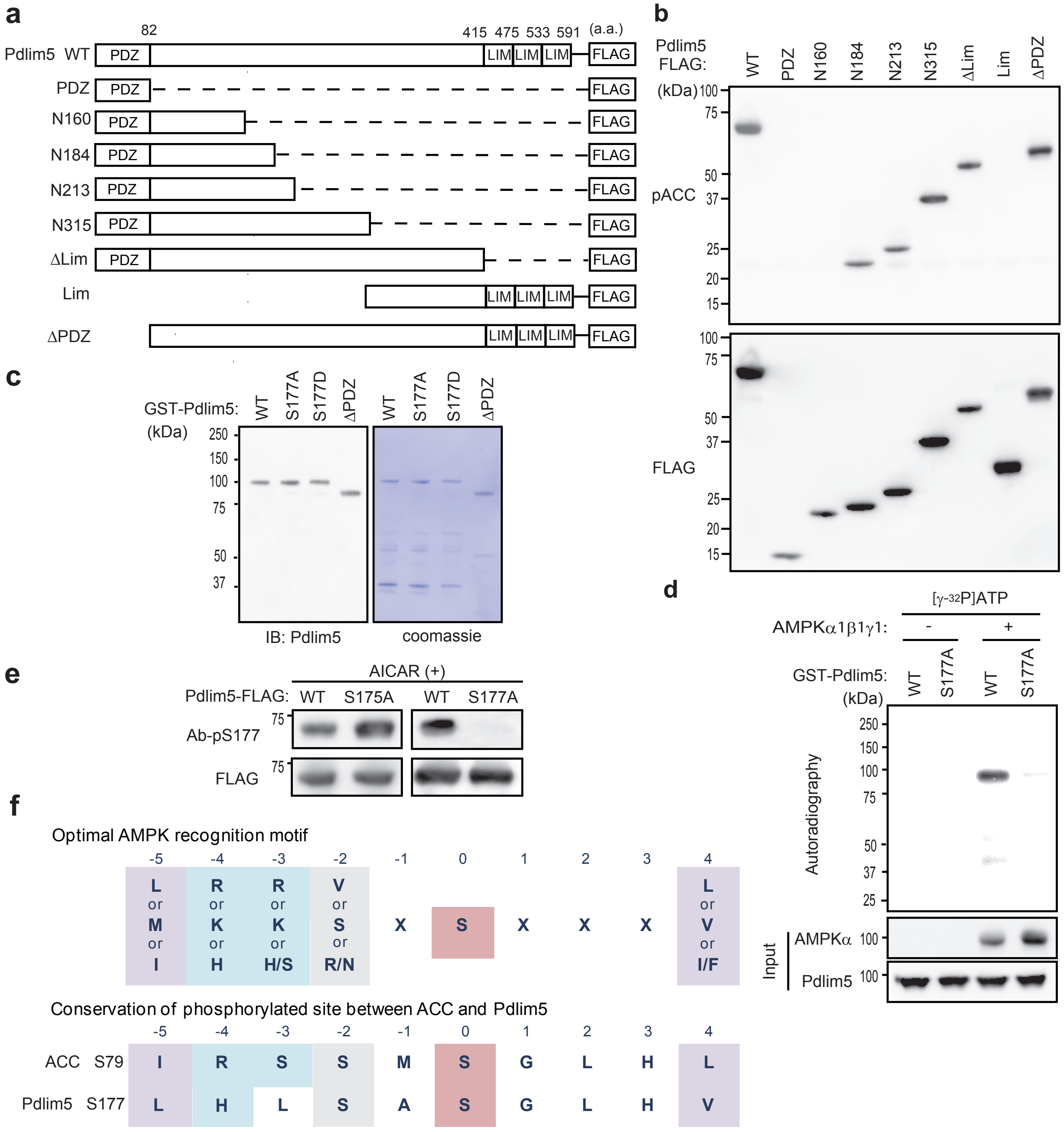

Supplementary Figure 2. Pdlim5 is directly phosphorylated at Ser177 by AMPK. (a) Diagrams of serially deleted Pdlim5 proteins. **(b)** HEK293T cells were transiently expressed with each deletion mutant of cFLAG-tagged Pdlim5, and then treated with AICAR (2 mM) for 15 min. Proteins purified using anti-FLAG M2 agarose were subjected to immunoblotting with pACC or anti-FLAG antibody. **(c)** Purified GST-tagged Pdlim5 (WT, S177A, and S177D) proteins. Images were obtained by immunoblotting with anti-Pdlim5 antibody (left panel) and Coomassie blue staining (right panel). **(d)** In vitro assay for AMPK phosphorylation. Recombinant GST-tagged Pdlim5 (WT or S177A) was incubated with baculovirus-expressed recombinant AMPK in the presence of [γ-^{32}P]ATP, and then subjected to autoradiography. **(e)** Validation of our original polyclonal antibody against S177-phosphorylated mouse Pdlim5 (Ab-pS177). HEK293T cells were transfected with cFLAG-tagged Pdlim5 (WT, S175A, and S177A) and treated with AICAR for 15 min. TCLs were subjected to immunoblotting with Ab-pS177. **(f)** Optimal AMPK recognition motif and amino-acid sequences of Pdlim5 and ACC surrounding the AMPK phosphorylation sites.

Supplementary Figure 3

Human_PDLim5	KVTSIPSPSSAFTPAHATTSSHASPSP-----VAAVTPPLFAA**S**GLHANANLSADQSPS
Chimp_PDLim5	KVTSIPSPSSAFTPAHATTSSHASPSP-----VAAVTPPLFAA**S**GLHANANLSADQSPS
Orangutan_PDLim5	KVTSIPSPSSAFTPAHATTSSRASPSP-----VAAVTPPQFAA**S**GLHANANLSADQSPS
Macaque_PDLim5	KVTSIPSPSSAFTPAHATTSSHASPSP-----VAAVTPPPFAA**S**GLHANANLSADQSPS
Dog_PDLim5	KVTSIPSPSSAFTPAHATISSHASPPP-----VAAVTPPSFAA**S**GLHANANLSADQHSS
Cow_PDLim5	KVTSIPSPSSAFTPAHATTSSHASPPP-----VAAVTSPPFAA**S**GPHANTNVSADQRSS
Pig_PDLim5	KVTSIPSPSSAFTPAHATTSSHASPPP-----VAAATPPPFAA**S**GLHANASLSADQRSS
Mouse_PDLim5	KVTSIPSPSSAFTPAHAATSSHASPTP-----VAAATPLHLSA**S**GLHVSANLSADQCSS
Rat_PDLim5	KVTSIPSPSSAFTPAHAATSSHASPPP-----VAAVTPPPLSA**S**GLHASANPSAAQCSS
Rabbit_PDLim5	KVTSIPSPSSAFTPAHAATSSHACPPP-----VAAVTPPPFAA**S**GLHVNANLSADQCSS
Dolphin_PDLim5	KVTSIPSPSSAFTPAHATTSSHASPAP-----GAAVTPPPFAA**S**GPHANASVSADQRSS
Chicken_PDLim5	KVTSIPSPSSAFTPAQAAP--------------MLPTPAPFAAPGLHVNAKPNADGWPP
Frog_PDLim5	KPASIPSASSAFTPASASLSIQSSPQPSALSLLAQVGPPPNSAPGLHANSKTTIEGHLP
Tetraodon_PDLim5	KVATIPSASSAFTPAAPSQAPQPPPNP------APAAPP--SHRRMASSSPSSSDSSSP

Supplementary Figure 3. Multiple sequence alignment of a region of Pdlim5 and putative orthologues. The alignment was generated using ClustalW. The serine at position 177 is shown in bold font.

Supplementary Figure 4

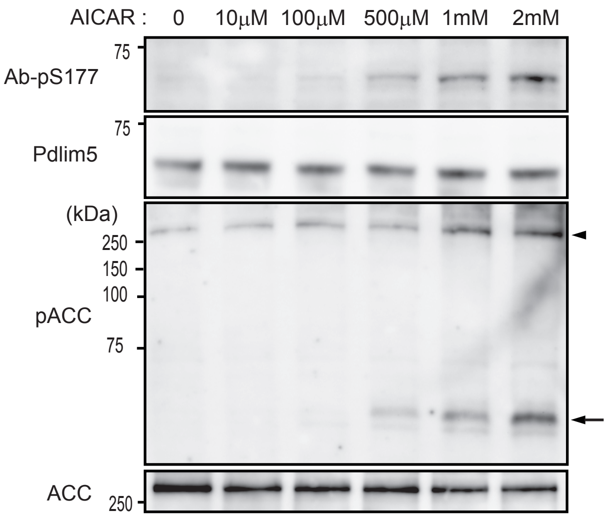

Supplementary Figure 4. Pdlim5 is phosphorylated upon treatment with AICAR in a dose-dependent manner. Vascular smooth muscle cells (vSMCs) were treated with the indicated concentrations of AICAR for 15 min. TCLs were subjected to immunoblotting with the indicated antibodies. Arrowhead indicates pACC, and arrow indicates signals from Ser177-phosphorylated Pdlim5. Phosphorylation of Pdlim5 is visible at AICAR concentrations of 500 μM and higher.

Supplementary Figure 5

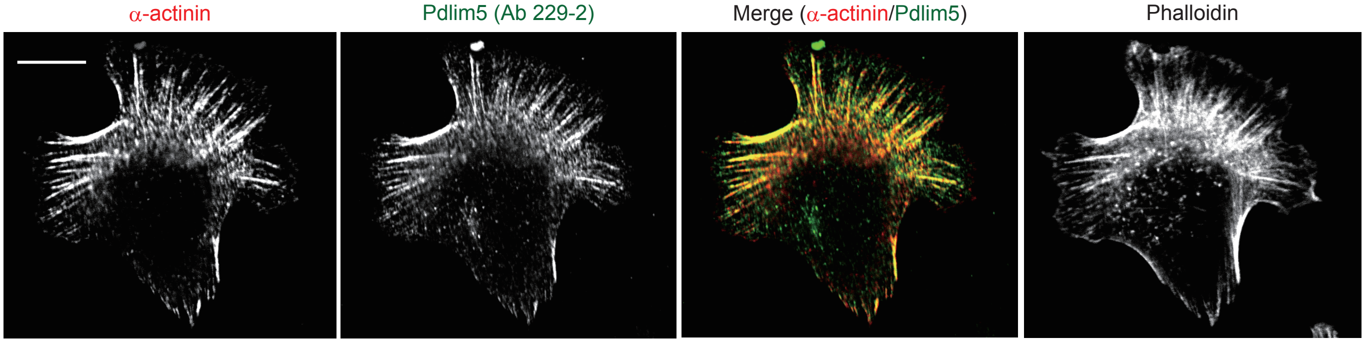

Supplementary Figure 5. Intracellular localization of endogenous Pdlim5. Immunostaining of vSMCs with anti-Pdlim5 antibody (Ab229-2), phalloidin, or α-actinin antibody. Scale bars, 10 μm.

Supplementary Figure 6

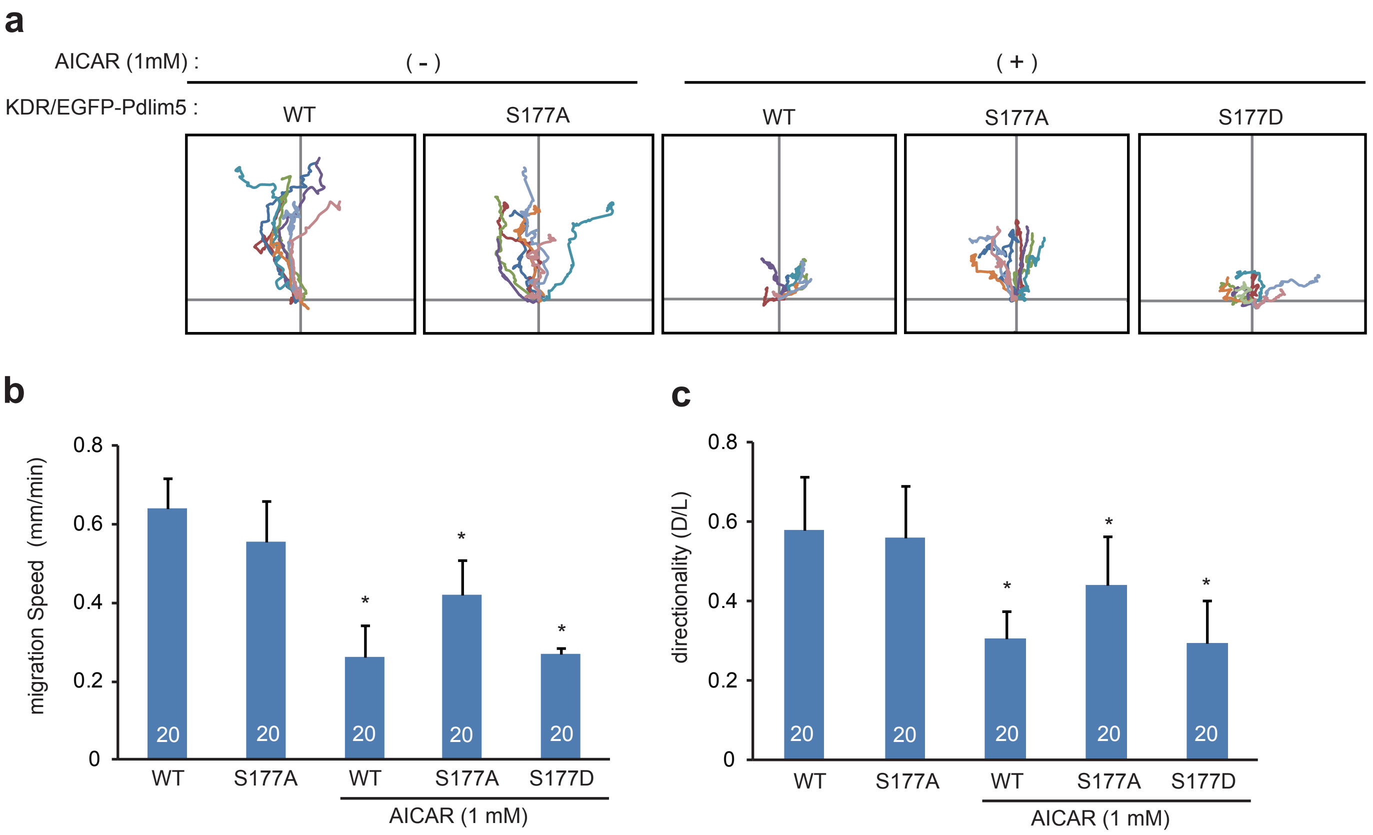

Supplementary Figure 6. AMPK activator–induced inhibition of cell migration was attenuated in S177A-Pdlim5–expressing cells. Scratch assays of KDR/EGFP-Pdlim5 (WT, S177A, and S177D) vSMCs were performed under treatment with AICAR (1 mM). Phase-contrast microscopy video of each group was taken for 8 h after scratching. **(a)** Analysis of migration paths over 8 h is shown. The origins of migration of each cell were superimposed at [0, 0]. **(b)** Bar graph showing migration speed of each cell [from (a)]. **(c)** Bar graph showing migration directionality of each cell [from (a)]. Numbers in the bars indicate *n*. Data are representative of means ± s.e.m from three independent experiments. Significance of differences between series of results was assessed using one-way ANOVA, followed by a post hoc comparison with Dunnett's method for multiple comparisons. $^*P <$ 0.01 compared to WT without AICAR treatment.

Supplementary Figure 7

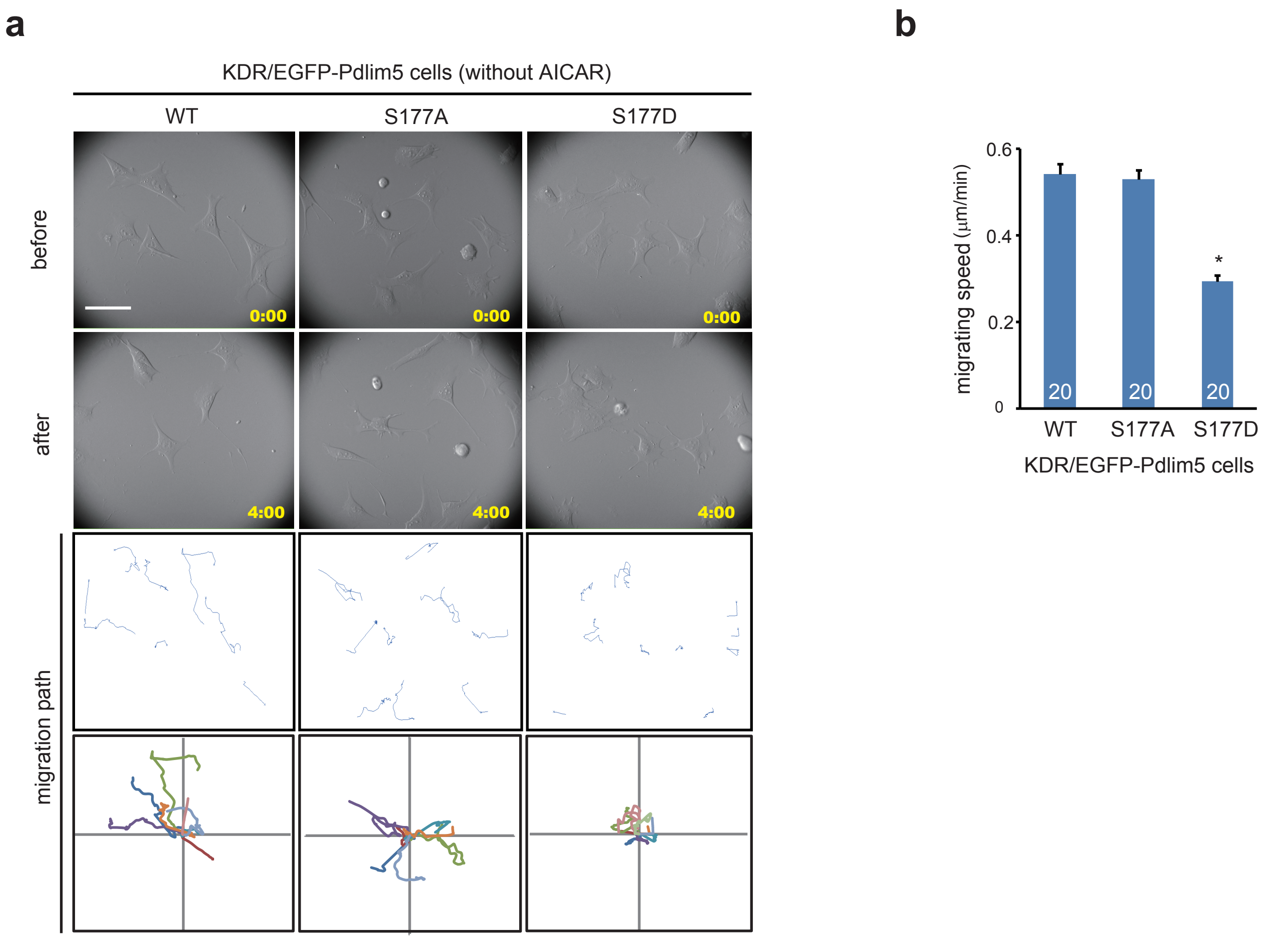

Supplementary Figure 7. Single cell migration assays. (a) Time-lapse DIC images of KDR/EGFP-Pdlim5 cells (WT, S177A, and S177D) in the absence of AICAR treatment. Images acquired before and after 4 h are shown. The lower middle row of each panel shows individual paths of migrating cells over 4 h. The bottom row of each panel shows analysis of migration paths. Tracks of representative cells shown in the lower middle row were plotted. The origins of migration were superimposed at [0, 0]. Scale bar, 30 μm. (b) Bar graph showing the migration speed of the cells [from (a)]; Numbers in the bars indicate *n*. Data are representative of means ± s.e.m from three independent experiments. Significance of differences between series of results was assessed using one-way ANOVA, followed by a post hoc comparison with Dunnett's method for multiple comparisons. *$P < 0.01$, compared to KDR/EGFP-WT-Pdlim5 cells.

Supplementary Figure 8

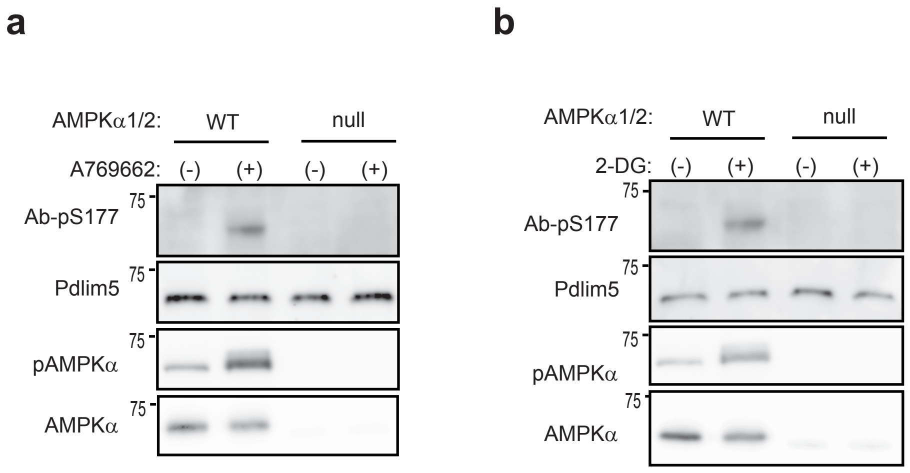

Supplementary Figure 8. Pdlim5 phosphorylation was blocked in AMPKα1/2-null MEFs. WT or AMPKα1/2-null MEFs were stimulated with A-769662 (100 μM) (a) or 2-DG (25 mM) (b) for 15 min. TCLs were subjected to immunoblotting with the indicated antibodies.

Supplementary Figure 9

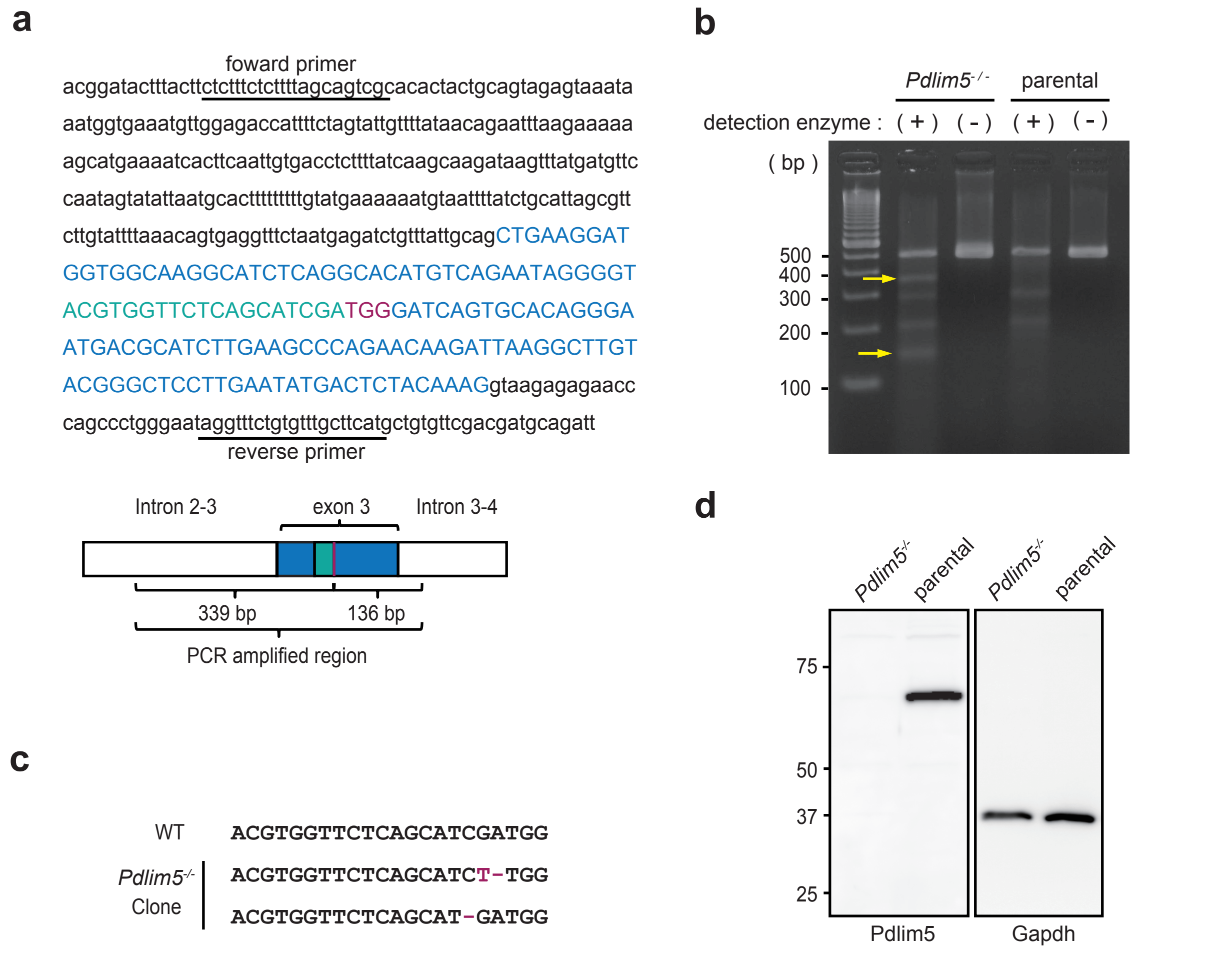

Supplementary Figure 9. Establishment of Pdlim5 knockout (*Pdlim5*$^{-/-}$) vSMC line. (a) Schematic representation of targeting sequences in exon 3 of the Pdlim5 gene. Exon 3 is highlighted in blue. The 19-bp target sequence of sgRNA is highlighted in turquoise, and protospacer adjacent motif (PAM) sequence is highlighted in magenta. Underlined sequences were used as PCR primers to amplify the region of interest. **(b)** Gel image of genomic cleavage detection assay. Genomic sequences at regions of interest were PCR-amplified from vSMCs transfected with a GeneArt CRISPR All-In-One vector targeting exon 3 of mouse Pdlim5 (*Pdlim5*$^{-/-}$ vSMCs) or parental vSMCs. After re-annealing, samples were treated with or without detection enzyme and run on a 2% agarose gel. The size of the parental band is 475 bp. Predicted cleaved bands of 339 bp and 136 bp (yellow arrows) appeared only in the lane of *Pdlim5*$^{-/-}$ vSMCs with detection enzyme. **(c)** Sequences of the target regions observed in the clone of *Pdlim5*$^{-/-}$ vSMCs. Magenta dashes and letters indicated the identified mutations. **(d)** Expression check of Pdlim5 protein in *Pdlim5*$^{-/-}$ vSMCs. TCLs from *Pdlim5*$^{-/-}$ vSMCs and parental vSMCs were subjected to immunoblotting with the indicated antibodies.

Supplementary Figure 10

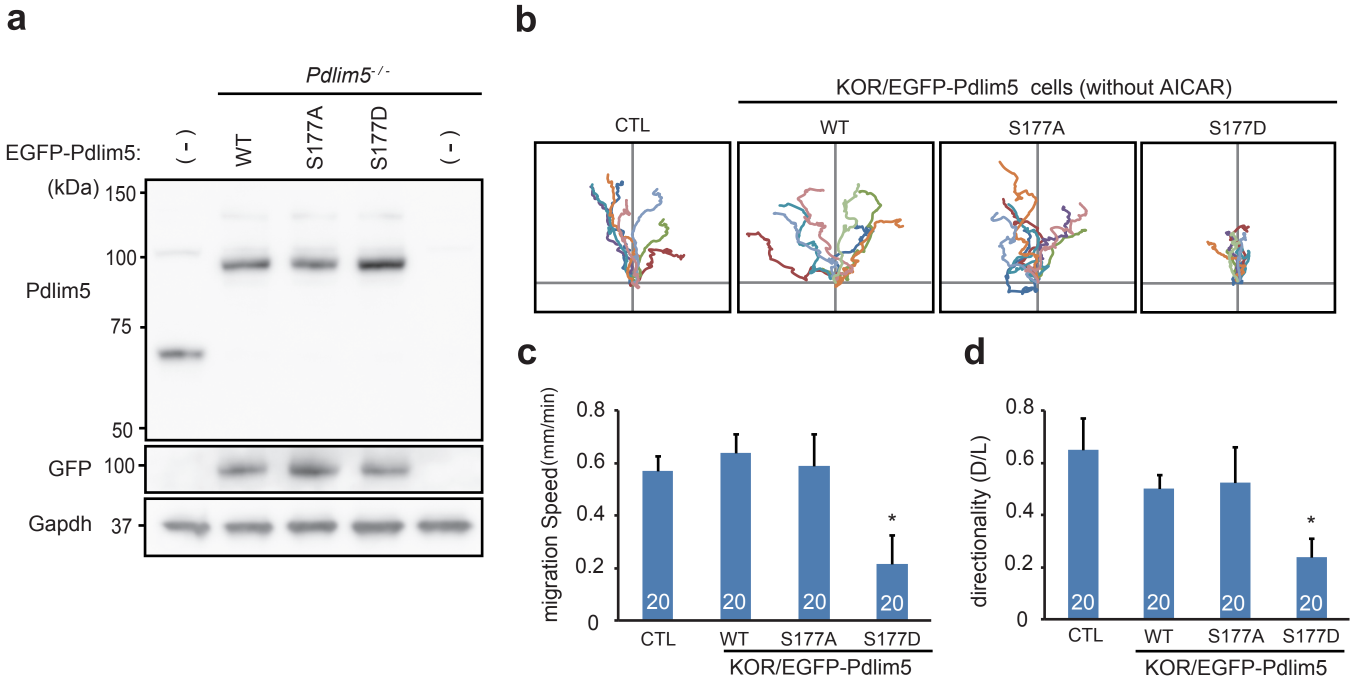

Supplementary Figure 10. Ser177 phosphorylation of Pdlim5 inhibited cell migration in the Pdlim5 knockout-and-rescue (KOR) vSMCs. (a) Establishment of the knockout and rescue (KOR) system for Pdlim5 in vSMCs. *Pdlim5*$^{-/-}$ vSMCs were transduced with EGFP-Pdlim5 (WT, S177A, or S177D) via adenoviral-mediated gene delivery. Total cell lysates (TCLs) from each group were subjected to immunoblotting with the indicated antibodies. **(b)** Scratch assay of KOR/EGFP-Pdlim5 (WT, S177A, and S177D) vSMCs. Phase-contrast microscopy video of each group was taken for 8 h after scratching in the absence of AICAR. Analysis of migration paths of each cell over 8 h. The origins of migration of each cell were superimposed at [0, 0]. **(c)** Bar graph showing migration speed of each cell [from (b)]. **(d)** Bar graph showing migration directionality of each cell [from (b)]. Numbers in the bars indicate *n*. Data are representative of means ± s.e.m from three independent experiments. Significance of differences between series of results was assessed using one-way ANOVA, followed by a post hoc comparison with Dunnett's method for multiple comparisons. **P* < 0.01 compared to WT.

Supplementary Figure 11

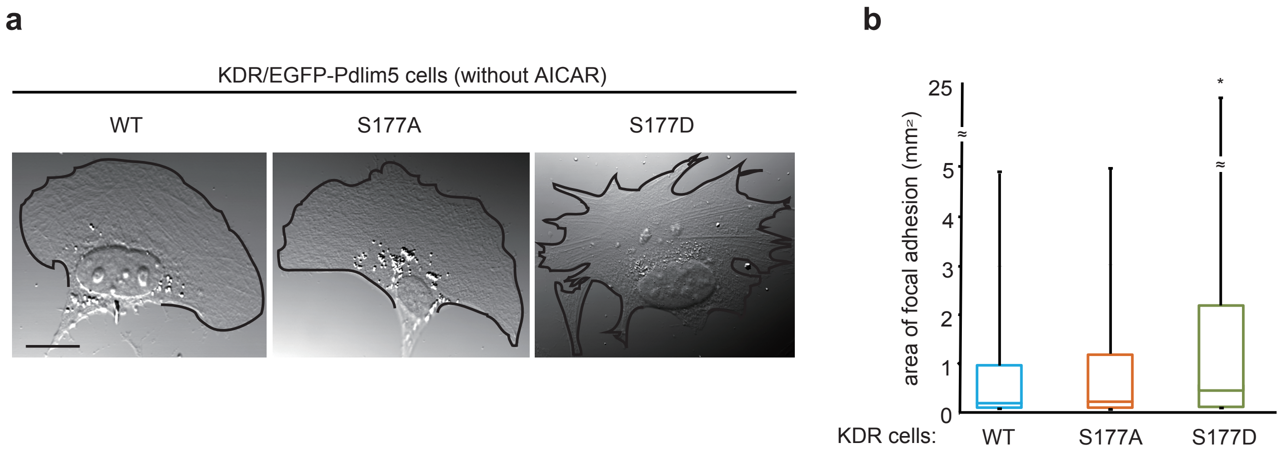

Supplementary Figure 11. Morphological changes in KDR cells. (a) DIC images of KDR/EGFP-Pdlim5 cells. Scale bar, 10 μm. **(b)** Box and whisker plots of the area stained with a paxillin antibody [from (Fig. 5a)] showing the 10th percentile (bottom line of each box), median (middle line of each box), 90th percentile (top line of each box), and the minimum and maximum values (each whisker). *n* = 576, 601, and 873 for WT, S177A, and S177D, respectively. Data are representative of means ± s.e.m from three independent experiments. Significance of differences between series of results was assessed using one-way ANOVA, followed by a post hoc comparison with Dunnett's method for multiple comparisons. **P* < 0.01 relative to KDR/EGFP-WT-Pdlim5 cells.

Supplementary Figure 12

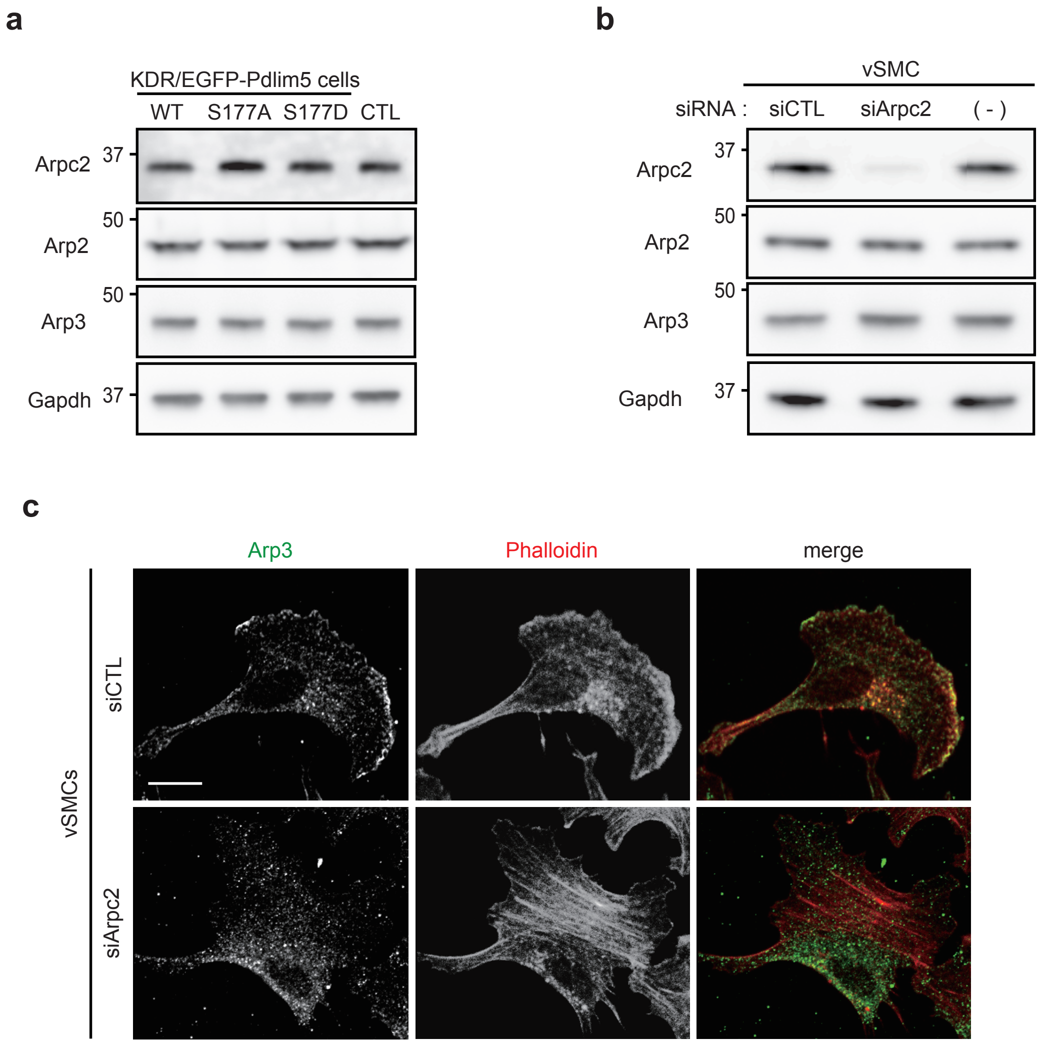

Supplementary Figure 12. Morphological changes in vSMCs treated with siRNA against Arpc2. **(a)** Expression level of endogenous Arpc2, Arp2, and Arp3, subunits of the Arp2/3 complex, in KDR/EGFP-Pdlim5 cells. TCLs from KDR cells were subjected to immunoblotting with the indicated antibodies. **(b)** TCLs from vSMCs treated with siRNA against Arpc2 were subjected to immunoblotting with the indicated antibodies. **(c)** Immunostaining of vSMCs defective for Arp2/3 complex due to siRNA against Arpc2. Cells were immunostained with anti-Arp3 antibody and phalloidin. Bars, 10 μm.

Supplementary Figure 13

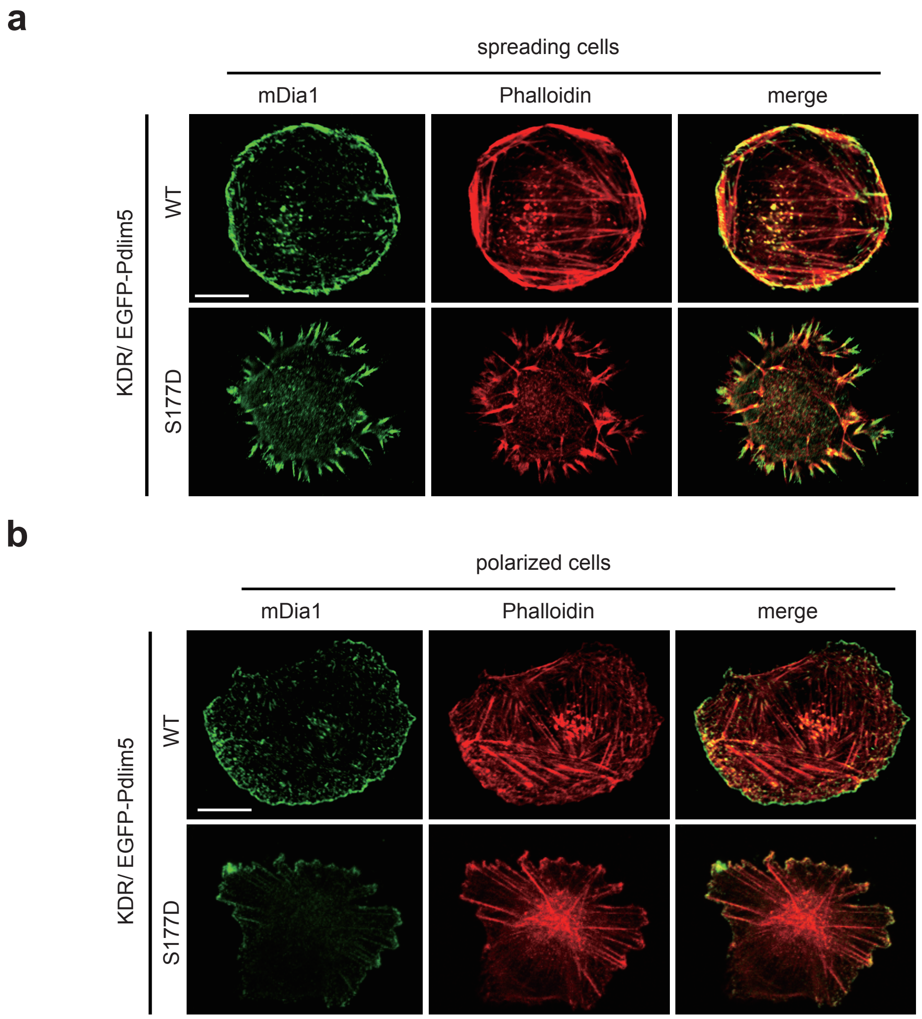

Supplementary Figure 13. Intracellular localization of mDia in KDR cells. (a and b) Immunostaining of spreading (a) or polarized (b) KDR/EGFP-Pdlim5 cells. Cells were fixed and stained with an mDia antibody and phalloidin. Bars, 10 μm.

Supplementary Figure 14

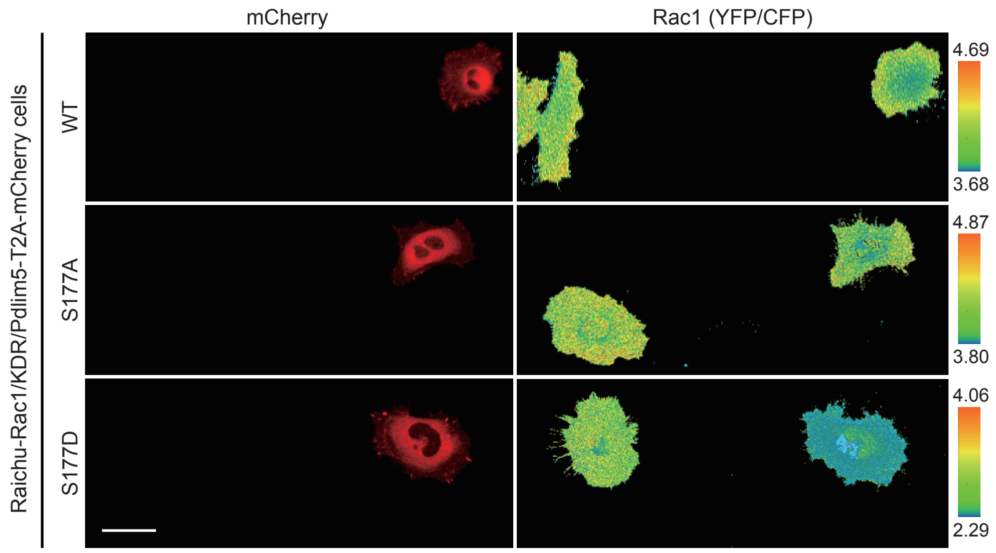

Supplementary Figure 14. Imaging of Rac1 activity. Each type of Raichu-Rac1/KDR/ Pdlim5-T2A-mCherry (WT, S177A, or S177D) cell was imaged for YFP and CFP. FRET efficiencies are shown as YFP/CFP ratio images. Bars, 20 μm.

Supplementary Figure 15

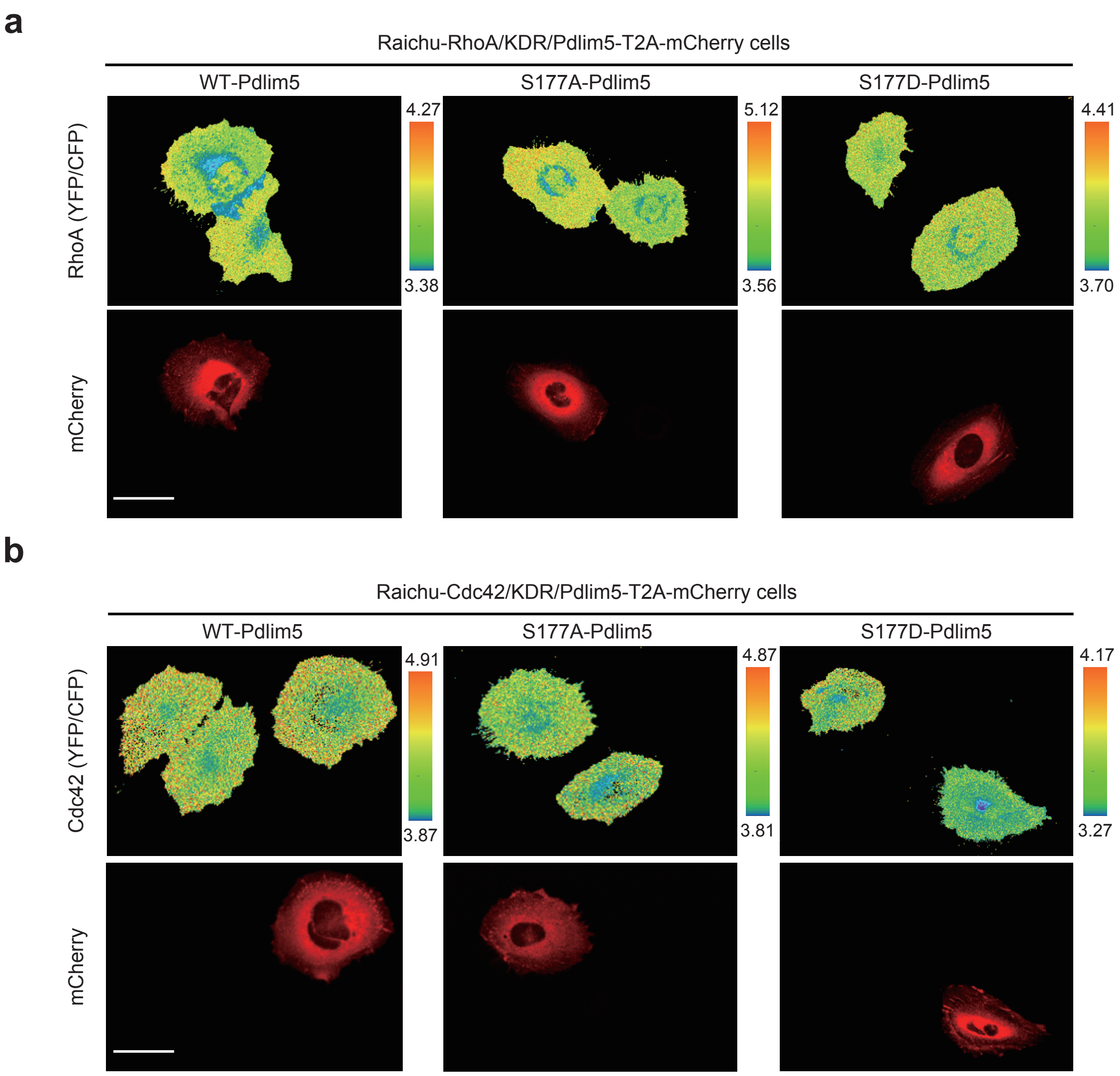

Supplementary Figure 15. Imaging of RhoA and Cdc42 activities. (a and b) Imaging of RhoA **(a)** and Cdc42 **(b)** activities. The KDR system was established in vSMCs stably expressing FRET probes specific for RhoA (Raichu-RhoA/vSMCs) or Cdc42 (Raichu-cdc42/vSMCs). Raichu-RhoA/vSMCs or Raichu-Cdc42/vSMCs were transfected with either siCTL or siPdlim5-2. siPdlim5-2–resistant Pdlim5-T2A-mCherry (WT, S177A, and S177D) was added via adenoviral-mediated gene delivery (Raichu-RhoA/ or Raichu-Cdc42/ KDR-Pdlim5-T2A-mCherry cells). Each cell was imaged for YFP and CFP. FRET efficiencies are shown as YFP/CFP ratio images. Bars, 20 μm.

Supplementary Figure 16

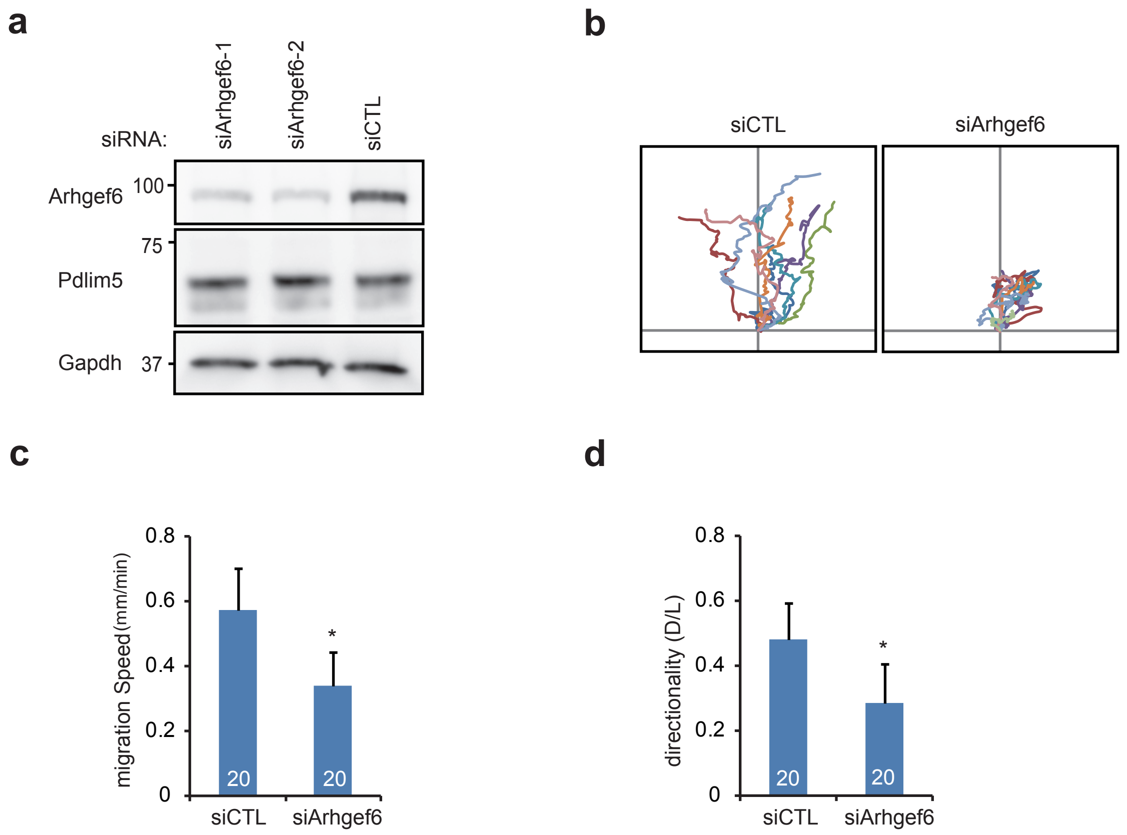

Supplementary Figure 16. vSMCs treated with siRNA against Arhgef6 exhibited disturbed cell migration. **(a)** Effects of siRNAs against Arhgef6. Cells were transfected with siRNAs, and TCLs were subjected to immunoblot analysis with the indicated antibodies. **(b)** Scratch assay of Arhgef6 knockdown vSMCs were performed. Phase-contrast microscopy video of each group was taken after scratching for 8 h. Analysis of migration paths over 8 h is shown. The origins of migration of each cell were superimposed at [0, 0]. **(c)** Bar graph showing migration speed of each cell [from (b)]. **(d)** Bar graph showing migration directionality of each cell [from (b)]. Numbers in the bars indicate *n*. Data are representative of means ± s.e.m from three independent experiments. Significance of differences between series of results was assessed using two-tailed unpaired Student's t-test. **P* < 0.01 compared with WT without AICAR treatment.

Supplementary Figure 17

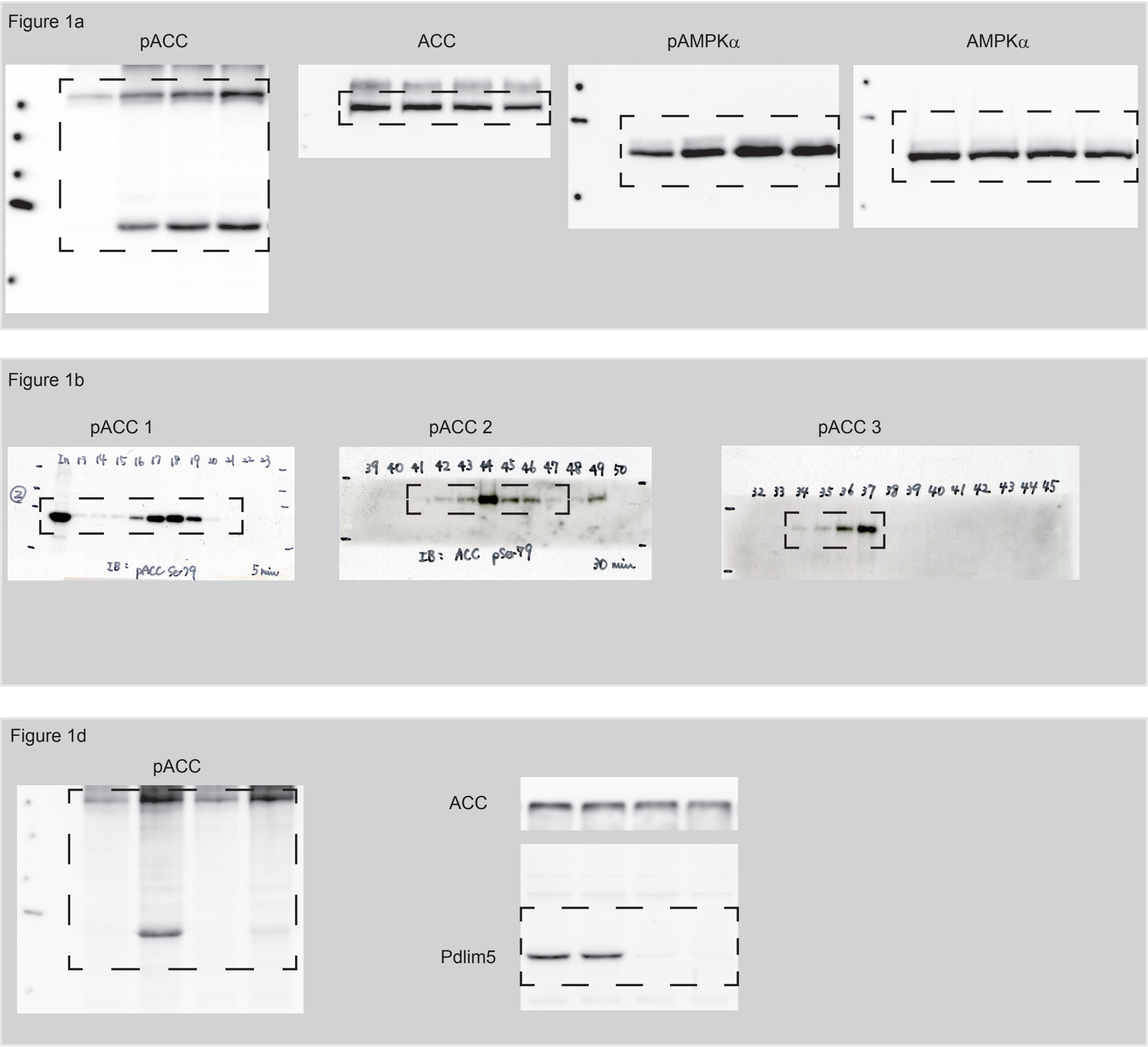

Supplementary Figure 17. Full scans of Western-blot data shown in Figure 1.
Rectangles delimit cropped areas used in the indicated panels in Figure 1.

Supplementary Figure 18

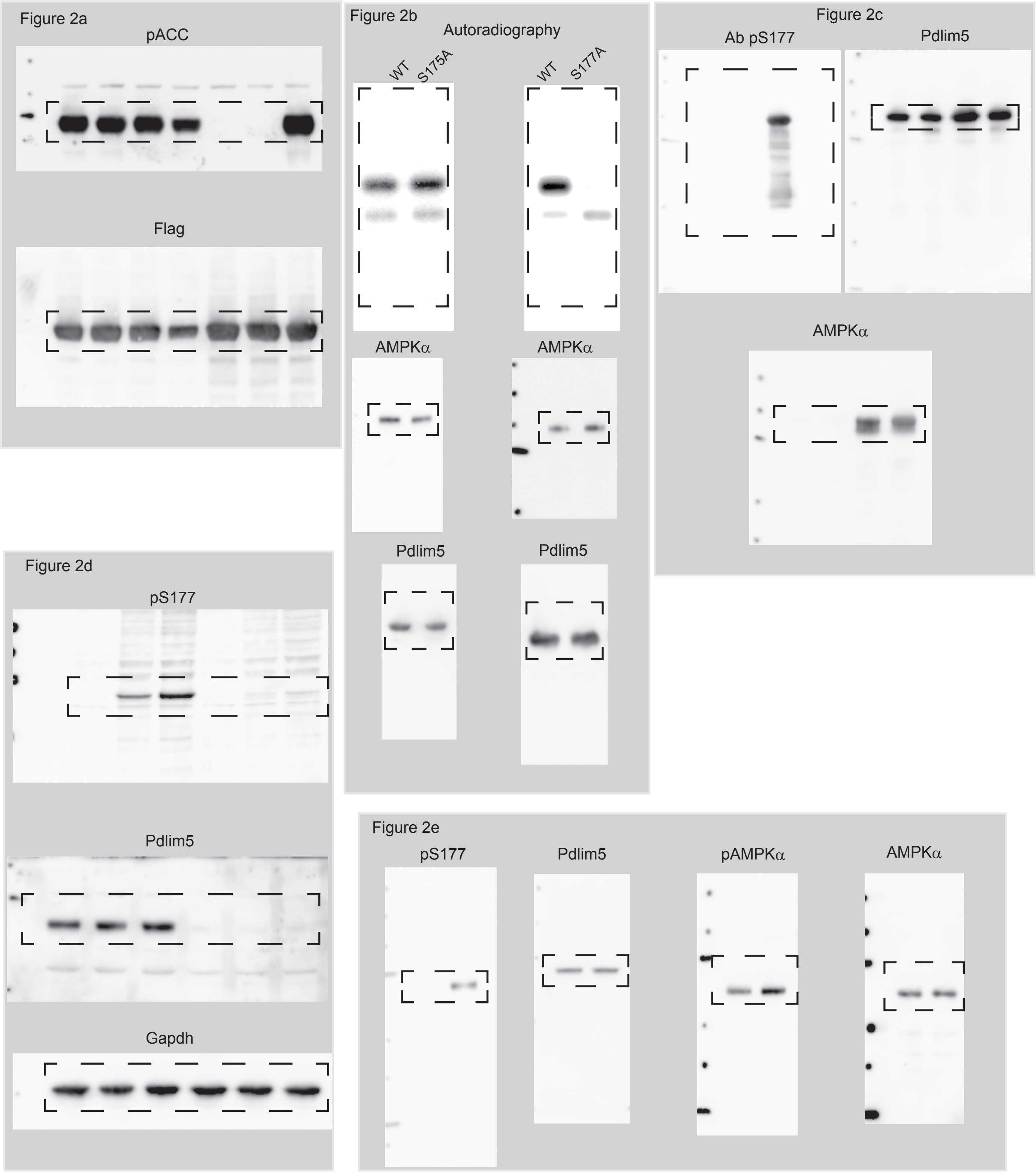

Supplementary Figure 18. Full scans of Western-blot data shown in Figure 2.
Rectangles delimit cropped areas used in the indicated panels in Figure 2.

Supplementary Figure 19

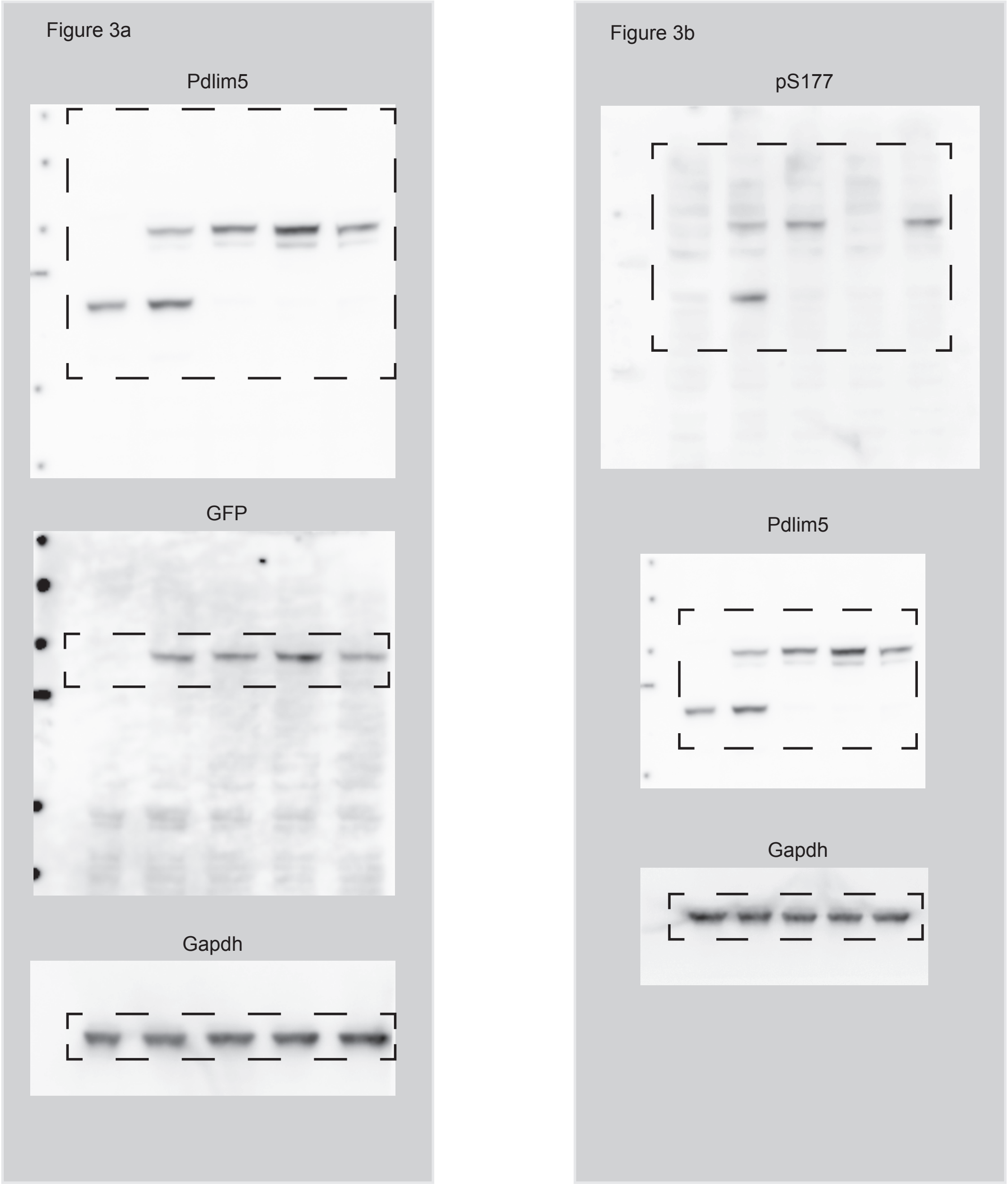

Supplementary Figure 19. Full scans of Western-blot data shown in Figure 3.
Rectangles delimit cropped areas used in the indicated panels in Figure 3.

Supplementary Figure 20

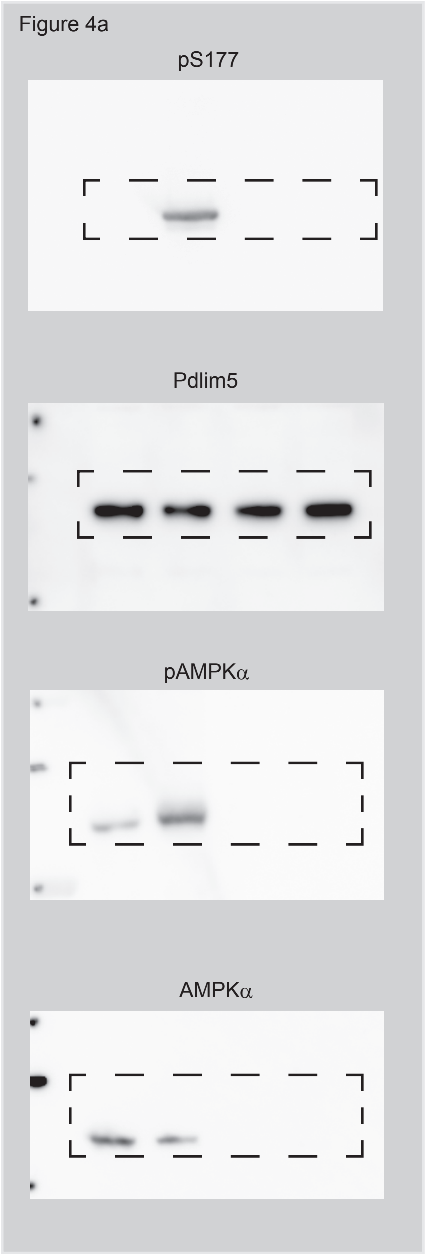

Supplementary Figure 20. Full scans of Western-blot data shown in Figure 4.

Rectangles delimit cropped areas used in the indicated panels in Figure 4.

Supplementary Figure 21

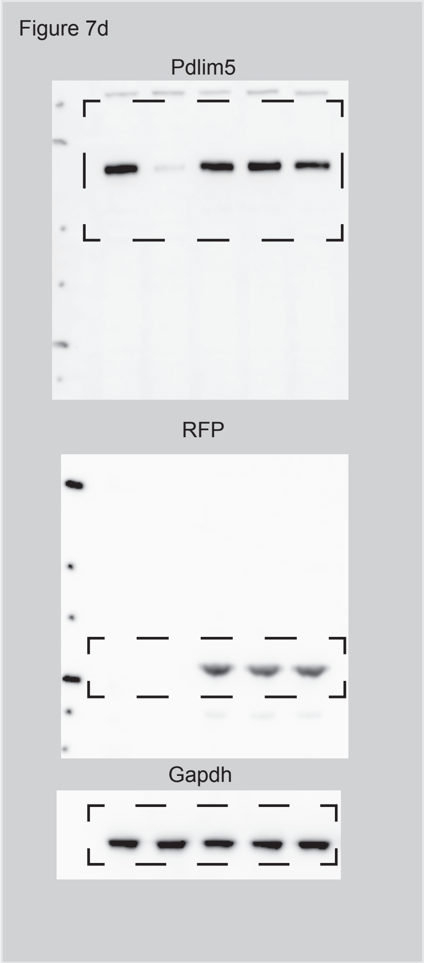

Supplementary Figure 21. Full scans of Western-blot data shown in Figure 7.
Rectangles delimit cropped areas used in the indicated panels in Figure 7.

Supplementary Figure 22

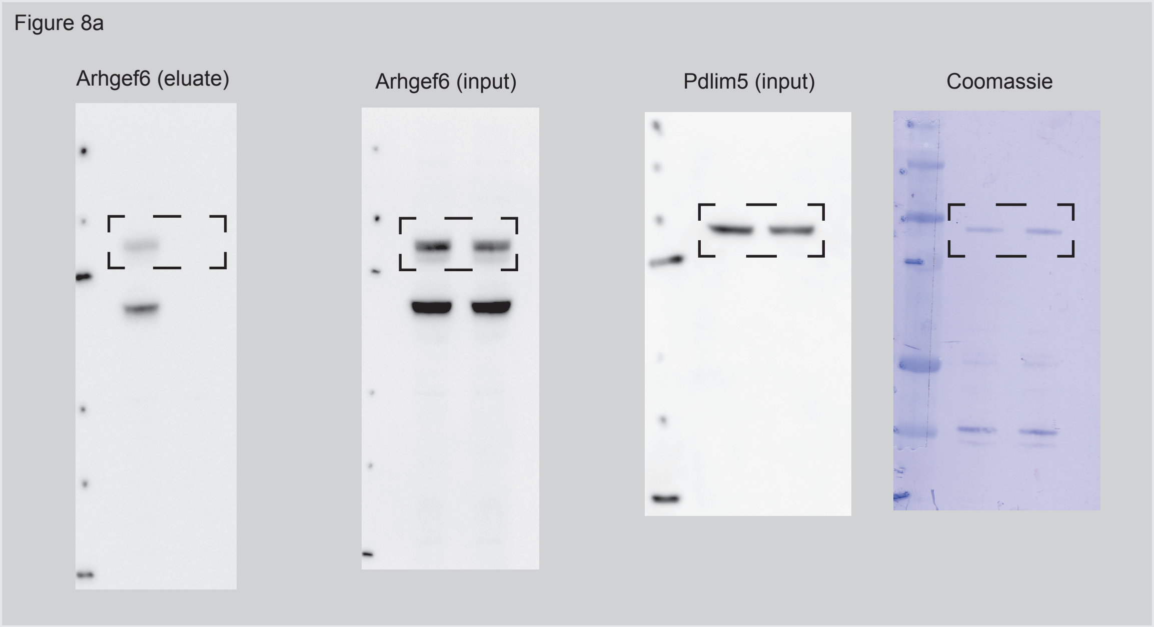

Supplementary Figure 22. Full scans of Western-blot data shown in Figure 8. Rectangles delimit cropped areas used in the indicated panels in Figure 8.

Supplementary Figure 23

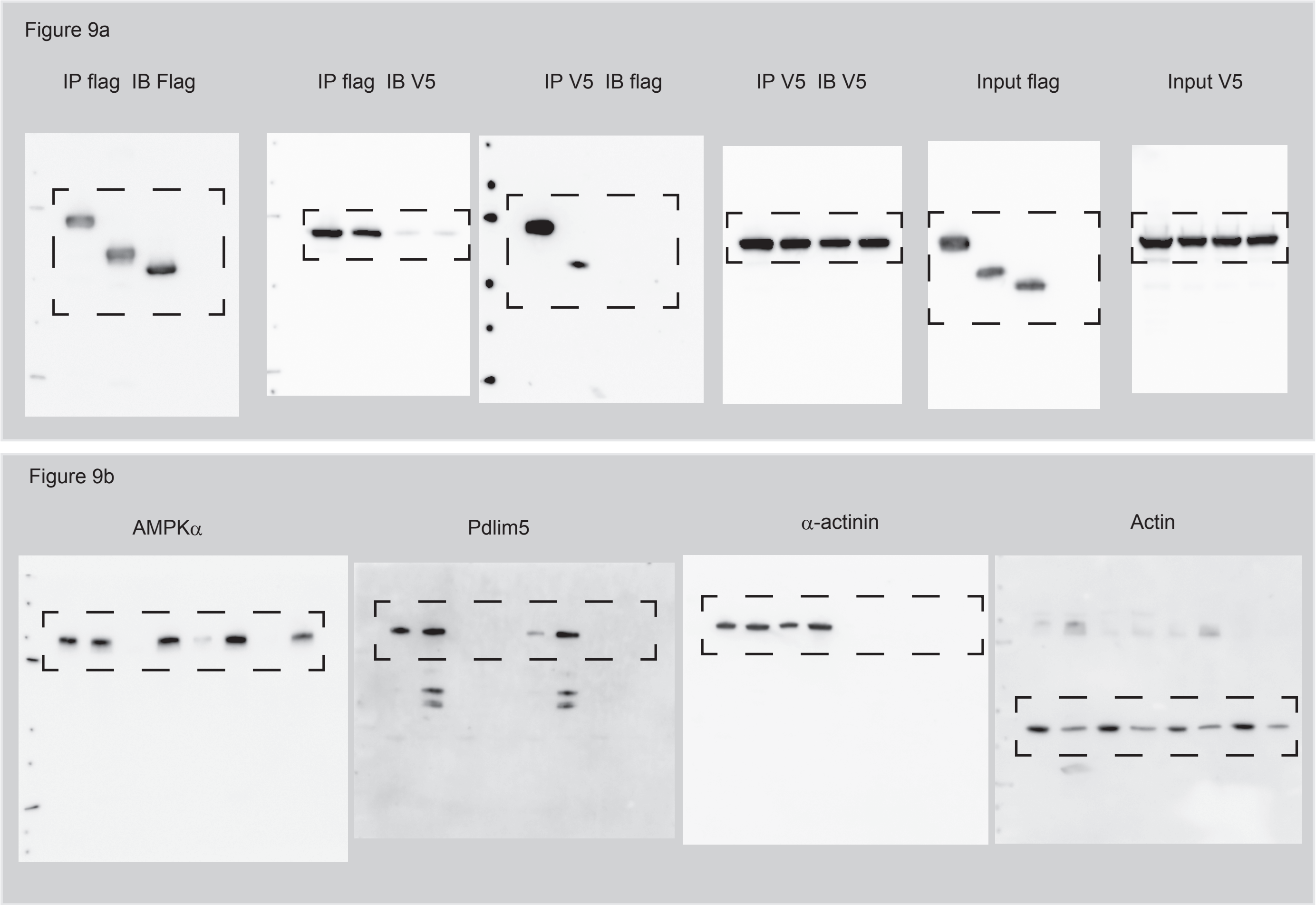

Supplementary Figure 23. Full scans of Western-blot data shown in Figure 9.
Rectangles delimit cropped areas used in the indicated panels in Figure 9.

Supplementary Table 1

List of RhoGEFs identified in the GST-Pdlim5 pull-down assay

Identified proteins	Identified peptide number	
Gene name	GST-PDZ WT	GST-PDZ S177D
PDLIM5 Isoform 1 of PDZ and LIM domain protein 5	6	6
ARHGEF2 Isoform 1 of Rho guanine nucleotide exchange factor 2	10	4
DOCK8 Isoform 1 of Dedicator of cytokinesis protein 8	6	2
DOCK2 Isoform 1 of Dedicator of cytokinesis protein 2	4	1
DOCK10 Isoform 1 of Dedicator of cytokinesis protein 10	2	0
ARHGEF6 Isoform 1 of Rho guanine nucleotide exchange factor 6	1	0
PARVG Isoform 1 of Gamma-parvin	1	0
ILK Integrin-linked protein kinase	1	0

GEFs are highlighted in bold.

Supplementary Methods

Plasmids

A cDNA fragment encoding full-length mouse Pdlim5 (NM_019808.3) was PCR amplified from mouse heart cDNA libraries and inserted into pENTR/D-TOPO using Gateway Technology (Invitrogen) to yield pENTR-WT Pdlim5. A set of cDNA fragments encoding amino acids 1–82 (PDZ), 1–160 (N160), 1–184 (N184), 1–213 (N213), 1–315 (N315), 1–415 (ΔLIM), 315–591 (LIM), and 82–591 (ΔPDZ) of mouse Pdlim5 were generated by PCR, using pENTR-WT Pdlim5 as a template, and inserted into pENTR/D-TOPO. A set of point mutants of mouse Pdlim5 in which Ser or Thr were replaced with Ala were generated by PCR, using pENTR-WT Pdlim5 as template and the following primer pairs: S162A, forward 5'-catgctgcccccacacctgtggc-3' and reverse 5'-ggtggaggggagtggcagcg-3'; T164A, forward 5'-cccgcacctgtggccgctgccactcccctc-3' and reverse 5'-ggaagcatgtgatgaagtggcggcat-3'; T170A, forward 5'-gctgccgctcccctccacctctctgcatc-3' and reverse 5'-tggcactaacatgcagtccg-3'; S175A, forward 5'-cacctcgctgcatccggactgcat-3' and reverse 5'-gcactaagattggcactaac-3'; S177A, forward 5'-gcagccggactgcatgttagtgccaatct-3' and reverse 5'-gagaggtggaggggagtggcagcggccaca-3'; S182A, forward 5'-catgttgctgccaatcttagtgc-3' and reverse 5'-gtggagatgaacactggtca-3' (underlined nucleotides indicate mutated sites). A phosphomimetic mutant of mouse Pdlim5 in which Ser177 was replaced with Asp was generated by PCR, using pENTR-WT Pdlim5 as template and the following primer pair: forward 5'-tgcagacgactgcatgttagtgccaatct-3' and reverse 5'-gagaggtggaggggagtggcagcggccaca-3'. To prepare FLAG-tagged proteins, pENTR-WT Pdlim5, pENTR-PDZ Pdlim5, pENTR-N160 Pdlim5, pENTR-N184 Pdlim5, pENTR-N213 Pdlim5, pENTR-N315 Pdlim5, pENTR-ΔLIM Pdlim5, pENTR-LIM Pdlim5, pENTR-ΔPDZ Pdlim5, pENTR-S162A Pdlim5, pENTR-S164A Pdlim5, pENTR-T170A Pdlim5, pENTR-S175A Pdlim5, pENTR-S177A Pdlim5, and pENTR-S182A Pdlim5 were subcloned into pEF-DEST51/cFLAG using the Gateway system to yield pEF-DEST51-WT Pdlim5, pEF-DEST51-PDZ Pdlim5, pEF-DEST51-N160 Pdlim5, pEF-DEST51-N184 Pdlim5, pEF-DEST51-N213 Pdlim5, pEF-DEST51-N315 Pdlim5, pEF-DEST51-ΔLIM Pdlim5, pEF-DEST51-LIM Pdlim5, pEF-DEST51-ΔPDZ Pdlim5, pEF-DEST51-S162A Pdlim5, pEF-DEST51-S164A Pdlim5, pEF-DEST51-T170A Pdlim5, pEF-DEST51-S175A Pdlim5, pEF-DEST51-S177A Pdlim5, and pEF-DEST51-S182A Pdlim5, respectively. To prepare V5-tagged protein, pENTR-WT AMPKα1 was subcloned into pEF-DEST51 vector using the Gateway system to yield pEF-DEST51-WT AMPKα1. To prepare the GST-fusion proteins, cDNA fragments encoding mouse WT-Pdlim5 and S177A-Pdlim5, S177D-Pdlim5, ΔPDZ-Pdlim5, and ΔLIM-Pdlim5 were subcloned into pGEX-6P-1 using the SalI and NotI restriction sites to yield pGEX-6P-1-WT Pdlim5, pGEX-6P-1-S177A Pdlim5, pGEX-6P-1-S177D Pdlim5, pGEX-6P-1-ΔPDZ Pdlim5, and pGEX-6P-1-ΔLIM Pdlim5, respectively. To prepare adenovirus vectors for expression of EGFP-tagged Pdlim5, cDNA fragments encoding mouse WT-Pdlim5, S177A-Pdlim5, and S177D-Pdlim5 were first subcloned into pEGFP-C1, in which a NotI site was introduced between the XhoI and EcoRI sites, using the XhoI and NotI sites, to yield pEGFP-C1-WT Pdlim5, pEGFP-C1-S177A Pdlim5, and pEGFP-C1-S177D Pdlim5, respectively. Next, cDNA fragments encoding EGFP-WT Pdlim5, EGFP-S177A Pdlim5, and EGFP-S177D Pdlim5 were amplified by PCR using pEGFP-C1-WT Pdlim5, pEGFP-C1-S177A Pdlim5, and pEGFP-C1-S177D Pdlim5 as templates, respectively, and inserted into pENTR/D-TOPO. Finally, the transgenes were subcloned into pAd/CMV/V5-DEST using the Gateway system. The PiggyBac transposon vector encoding the FRET biosensor for Rac1/RhoA/Cdc42 and the blasticidin resistance gene were generous gifts from Dr. Mochizuki (National Cerebral and Cardiovascular Center Research Institute, Japan). pCMV-hyPBase vector encoding PiggyBac transposase was obtained from Wellcome Trust Sanger Institute.

Establishment of Pdlim5-knockout vSMCs (*Pdlim5*$^{-/-}$ vSMCs)

We performed CRISPR/Cas9-induced genome editing to make the *Pdlim5*$^{-/-}$ vSMCs line using the GeneArt CRISPR Nuclease Vector Kit (Life Technologies). Briefly, we chose a target sequence on exon 3 of the Pdlim5 gene (Supplementary Fig. 9) and designed single-stranded oligonucleotide primers encoding target-specific crRNA: top-strand oligo, 5'-ACGTGGTTCTCAGCATCGATGTTTT-3'; bottom-strand oligo, 5'-ATCGATGCTGAGAAC CACGTCGGTG-3'. After annealing equal amounts of each single-stranded oligonucleotide, the resulting double-stranded oligonucleotide was cloned into the GeneArt CRISPR Nuclease CD4 vector (Pdlim5 guide CRISPR Nuclease CD4 vector). vSMCs grown in a 100-mm dish were transfected with 15 μg of Pdlim5 guide CRISPR Nuclease CD4 vector using Lipofectamine 3000 (Life Technologies). Cell were trypsinized 3 days after the transfection, resuspended in PBS with 0.1% BSA and 2 mM EDTA, and then cells expressing Cas9 and gRNA were isolated and enriched using the Dynabeads CD4 Positive Isolation Kit (Invitrogen). Isolated CD4-positive cells were isolated in 96-well plates, and clonal cell populations were expanded. Pdlim5 protein expression of each clone was examined by immunoblotting against Pdlim5. Among them, clone B2 presented with a complete absence of Pdlim5 protein and used for further studies. Genomic DNA was extracted from 1×10^6 cells, and locus-specific cleavage was analyzed using the GeneArt Genomic Cleavage Detection Kit (Life Technology). Target genomic DNA sequence was PCR amplified using the following primers: forward, CTCTTTCTCTTTTAGCAGTCGC; reverse, GCATGAAGCAAACACAGAAACC.
